# Tropical Ehrhart theory and tropical volume

**DOI:** 10.1007/s40687-020-00228-1

**Published:** 2020-09-21

**Authors:** Georg Loho, Matthias Schymura

**Affiliations:** 1 London School of Economics and Political Science, Houghton Street, London, WC2A 2AE UK; 2 BTU Cottbus-Senftenberg, Platz der Deutschen Einheit 1, 03046 Cottbus, Germany

**Keywords:** Tropical lattices, Ehrhart theory, Tropical volumes, Tropical polytopes, Tropical semiring, Tropical geometry, 14T05, 52C07, 52A38

## Abstract

We introduce a novel intrinsic volume concept in tropical geometry. This is achieved by developing the foundations of a tropical analog of lattice point counting in polytopes. We exhibit the basic properties and compare it to existing measures. Our exposition is complemented by a brief study of arising complexity questions.

## Introduction

Tropical geometry is the study of piecewise-linear objects defined over the $(\max ,+)$-semiring that arises by replacing the classical addition ‘$+$’ with ‘$\max$’ and multiplication ‘$\cdot$’ with ‘$+$.’ While this often focuses on combinatorial properties, see [11, 25], we are mainly interested in metric properties. Measuring quantities from tropical geometry turned out to be fruitful for a better understanding of interior point methods for linear programming [2] and principal component analysis of biological data [37]. Moreover, it has interesting connections with representation theory [29, 38] and computational complexity [22].

Driven by this motivation, we develop a new definition of a volume for tropical convex sets by a thorough investigation of the tropical analog of lattice point counting. This continues the investigation of intrinsic tropical metric properties that started around a tropical isodiametric inequality [15] and tropical Voronoi diagrams [14].

Tropical polytopes are finitely generated tropical convex sets, see (2) in Sect. 2.1. Former work only considered the lattice points ${\mathbb {Z}}^d$ in a *d*-dimensional polytope, see in particular [12]. This idea was used to measure its *Euclidean volume* and deduce the hardness to compute it by counting the integer lattice points [22]. These lattice points arise naturally through the representation of affine buildings as tropical polytopes [29]. However, we are more interested in lattice points which are conformal with the semiring structure. Varying the semiring as explained in Sect. 2.3 leads to two natural notions: integer lattice points in polytopes over the $(\max , \cdot )$-semiring and their image under a logarithm map over the $(\max ,+)$-semiring. This is related to the concept arising from ‘dequantization,’ but we show in Sect. 4.3 how our tropical volume concept differs from the existing ones [15].

The main idea leading to our novel concept of tropical volume is the following: For a classical polytope $P \subseteq {\mathbb {R}}^d$, the Euclidean volume describes the asymptotic behavior of its *Ehrhart function* $L(P,k) = \#\,\left( kP \cap {\mathbb {Z}}^d\right)$, that is, the function that counts lattice points from ${\mathbb {Z}}^d$ that are contained in the *k*th dilate of the polytope *P*. This discretization further refines if *P* is a *lattice polytope*, meaning that all its vertices belong to ${\mathbb {Z}}^d$. In fact, Ehrhart proved that in this case *L*(*P*, *k*) agrees with a polynomial of degree at most *d*, for every positive integral dilation factor $k \in {\mathbb {Z}}_{>0}$ (see [6, Ch. 3]):

$$\begin{aligned} L(P,k) = \sum _{i=0}^d c_i(P) k^i. \end{aligned}$$

The polynomial on the right-hand side is known as the *Ehrhart polynomial* of *P*, and the crucial point for us is that

$$\begin{aligned} {{\,\mathrm{vol}\,}}(P) = \lim _{k \rightarrow \infty } \frac{L(P,k)}{k^d} = c_d(P). \end{aligned}$$

Now, our approach toward an intrinsic tropical volume concept is to turn this discretization process around and to establish tropical analogs to the previously described classical ideas. This will be done in four steps:

(i) We define a suitable concept of tropical lattice (depending on a fineness parameter) and tropical lattice polytopes in Sect. 2.2.
(ii) In Sect. 3, we develop a tropical Ehrhart theory showing that the corresponding tropical Ehrhart function exhibits polynomial behavior.
(iii) We then take the leading coefficient of the tropical Ehrhart polynomial as the definition of tropical volume.
(iv) Finally, we extract the metric information that is independent of the fineness parameter of the tropical lattice by using its asymptotics and extend it to all tropical polytopes, without any integrality restriction. This is implemented in Sect. 4.

The development of our tropical Ehrhart theory rests on making the transition from the ring $({\mathbb {R}},+,\cdot )$ to the tropical semiring ${\mathbb {T}}= ({\mathbb {R}}\cup \{-\infty \},\max ,+)$ in two steps. More precisely, we first replace addition ‘+’ by the maximum operation to obtain the semiring $S_{(\max ,\cdot )} = ({\mathbb {R}}_{\ge 0},\max ,\cdot )$. Then, for any $b \in {\mathbb {N}}_{\ge 2}$, the map $x \mapsto \log _b(x)$ induces a semiring isomorphism between $S_{(\max ,\cdot )}$ and ${\mathbb {T}}$.

On the one hand, this point of view motivates us to introduce tropical integers as $\log _b({\mathbb {Z}}_{\ge 0})$, leading to what we call the *tropical* *b*-*lattice* $\log _b({\mathbb {Z}}_{\ge 0})^d$ with fineness parameter $b \in {\mathbb {N}}_{\ge 2}$. And on the other hand, it allows to transfer classical Ehrhart theory on complexes of lattice polytopes to an Ehrhart theory for lattice polytopes over the various semirings which we explicitly describe in Theorems 3.2, 3.4, and 3.6. These results heavily rely on the interplay of the involved semirings associated with tropical geometry, cf. [11]. While this approach is very conceptual and offers a first understanding of tropical Ehrhart theory, it has the disadvantage of lacking a useful description of the coefficients of the resulting tropical Ehrhart polynomials.

Therefore, we take a second route based on the covector decomposition that allows to triangulate a tropical lattice polytope into so-called alcoved simplices which are both tropically and classically convex polytopes. This leads to the explicit representations of tropical Ehrhart coefficients in Theorem 3.14 and eventually to our desired intrinsic volume concept. The key insight here is that counting tropical lattice points in tropical dilations of alcoved simplices amounts to counting usual lattice points in dilates of diagonally transformed alcoved simplices (Lemma 3.11). To assemble the Ehrhart coefficients correctly from these pieces, we need a better understanding of lower-dimensional structures of the covector decomposition, which is achieved in Sect. 2.1.

As the result of the four-step-process outlined above, we define the *tropical barycentric volume* ${{\,\mathrm{tbvol}\,}}(P)$ of a tropical polytope $P \subseteq {\mathbb {T}}^d$ as

$$\begin{aligned} {{\,\mathrm{tbvol}\,}}(P) := \max _x \, (x_1+\cdots +x_d), \end{aligned}$$

where the maximum is taken over all points $x \in P$ that are contained in a *d*-dimensional cell of the polyhedral complex associated to *P*. Our choice of name will become clear later on.

In Sect. 4.2, we investigate basic properties of the tropical barycentric volume. We prove that it satisfies the natural tropical analogs of the fundamental properties of the Euclidean volume: monotonicity, the valuation property, rotation invariance, homogeneity, non-singularity, and multiplicativity. In this sense, ${{\,\mathrm{tbvol}\,}}(\cdot )$ is a meaningful and intrinsic volumetric concept for tropical geometry.

Furthermore, in Sect. 4.3 we compare the tropical barycentric volume with existing volumetric measures. For instance, it turns out to be bounded by the tropical dequantized volume ${{\,\mathrm{qtvol}\,}}^+(\cdot )$ defined in [15]. More precisely, if $P = {{\,\mathrm{tconv}\,}}(M)$ is the tropical polytope defined as the tropical convex hull of the columns of $M \in {\mathbb {T}}^{d \times m}$, then we prove in Theorem 4.15 that

1 $$\begin{aligned} {{\,\mathrm{tbvol}\,}}(P) \le {{\,\mathrm{qtvol}\,}}^+(M). \end{aligned}$$

Motivated by this inequality, we go a step further and work toward lower-dimensional volumetric measures in Sect. 5. We propose natural generalizations of the tropical barycentric volume that may serve as adequate tropical versions of the classical intrinsic volumes (or quermassintegrals) (cf. [35]). For example, we define a tropical lower barycentric *i*-volume ${{\,\mathrm{tbvol}\,}}_i^-(P)$ of $P = {{\,\mathrm{tconv}\,}}(M)$ and prove that it is upper bounded by the maximal tropical determinant of an $(i \times i)$-submatrix of *M* (see Theorem 5.12). This extends (1), because ${{\,\mathrm{qtvol}\,}}^+(M)$ can be defined as the maximal tropical determinant of a $(d \times d)$-submatrix of *M*; see [15].

We close the paper with Sect. 6 in which we discuss computational aspects of the problem of computing the tropical barycentric volume. We argue that the decision problem that asks whether the tropical barycentric volume of a given tropical polytope is nonvanishing is equivalent to checking feasibility of a tropical linear program or to deciding winning positions in mean-payoff games. Therefore, this decision problem lies in NP $\cap$ coNP (cf. [22]). This equivalence is analogous to the classical setting, where existence of interior points in a polytope is equivalent to solving linear programs (cf. [24]). Based on the computation of the tropical barycenter of a tropical simplex, we moreover devise an algorithm to determine the tropical barycentric volume of a tropical *d*-polytope with *m* vertices, that runs in time $$O(\left( {\begin{array}{c}m\\ d+1\end{array}}\right) d^3)$$.

## Tropical convexity and tropical lattices

In this section, we fix the main notation of the paper, discuss the crucial concept of the *i*-*trunk* of a tropical polytope, introduce the notion of tropical lattice leading to our tropical Ehrhart theory, and finally review the relationship between different versions of convexity relevant to our studies.

### Tropical polytopes and alcoved triangulations

We denote by ${\mathbb {T}}= ({\mathbb {R}}\cup \{-\infty \}, \oplus , \odot )$ the *max-tropical semiring*, where $\oplus$ denotes the $\max$ operation and $\odot$ denotes the classical addition ‘$+$.’ The *tropical convex hull* of a set $V \subseteq {\mathbb {T}}^d$ is defined by

2 $$\begin{aligned} {{\,\mathrm{tconv}\,}}(V) = \bigg \{\bigoplus _{j=1}^{n} \lambda _j \odot v_j : \lambda _1,\ldots ,\lambda _n \in {\mathbb {T}}, \bigoplus _{j=1}^n \lambda _j = 0, v_1,\ldots ,v_n \in V \bigg \} . \end{aligned}$$

If *V* is finite, this is called a *tropical polytope*. We will switch freely between matrices and the set of their columns. A set is *tropically convex* if it contains the tropical convex hull of each of its finite subsets. By the tropical Minkowski–Weyl theorem [21], there is a unique minimal set of points generating a tropical polytope; we call these points the *vertices*.

**Fig. 1:**
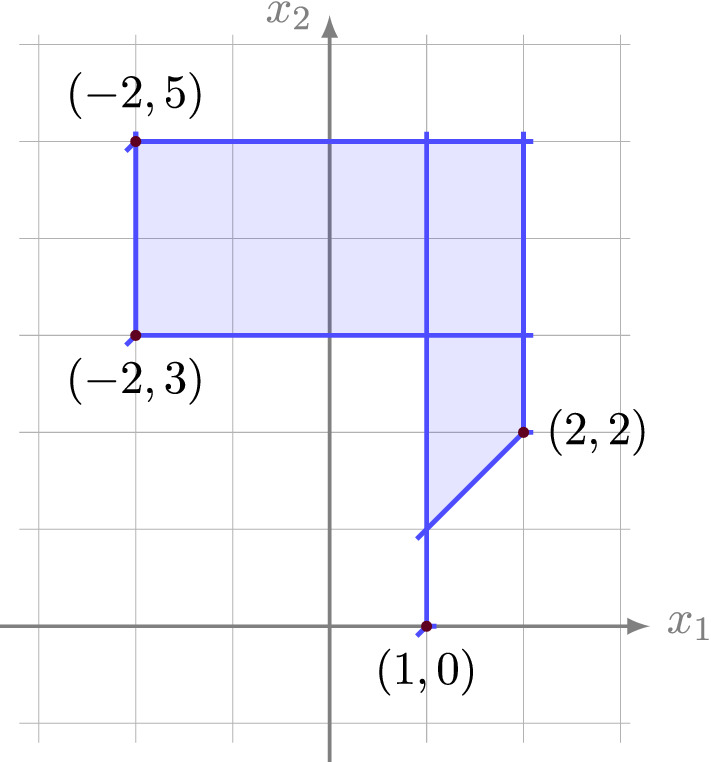
The covector decomposition of a tropical 2-polytope. It consists of three quadrilaterals and a line segment, and it has nine pseudovertices. Its 2-trunk is obtained by cutting off the line segment connecting (1, 1) to (1, 0)

The ‘type decomposition’ due to Develin and Sturmfels [17] shows that each tropical polytope has a decomposition into *polytropes*, which are classically and tropically convex polytopes [27]. Following [20], we use the name *covector decomposition* for this polyhedral complex formed by the polytropes. The vertices of the covector decomposition are called *pseudovertices* and the *dimension* of the tropical polytope is the maximal dimension of a polytope in the complex. Figure 1 depicts the covector decomposition of a tropical 2-polytope with vertices $\{(1,0)^\intercal ,(2,2)^\intercal ,(-2,3)^\intercal ,(-2,5)^\intercal \}$.

For a tropical polytope $P \subseteq {\mathbb {T}}^d$, let the family of relatively open polytopes in the covector decomposition of *P* be denoted by ${\mathcal {F}}_P$. An element $T \in {\mathcal {F}}_P$ is called an *i*-*tentacle element*, if it is not contained in the closure of any $(i+1)$-dimensional polytope $Q \in {\mathcal {F}}_P$. In particular, the dimension of an *i*-tentacle element is smaller than or equal to *i*. The following subcomplexes of ${\mathcal {F}}_P$ will be important later on and thus deserve some initial studies.

#### Definition 2.1

(*i*-*trunk*) Let *P* be a tropical polytope and let $i \in \{1,\ldots ,d\}$. We define the *i*-*trunk* of *P* as

$$\begin{aligned} {{\,\mathrm{Tr}\,}}_i(P) := \bigcup \left\{ F \in {\mathcal {F}}_P : \exists \, G \in {\mathcal {F}}_P\text { with }\dim (G) \ge i\text { such that }F \subseteq G \right\} . \end{aligned}$$

This means, that we obtain ${{\,\mathrm{Tr}\,}}_i(P)$ from *P* after removing every $(i-1)$-tentacle element. We always have $P = {{\,\mathrm{Tr}\,}}_1(P) \supseteq {{\,\mathrm{Tr}\,}}_2(P) \supseteq \cdots \supseteq {{\,\mathrm{Tr}\,}}_d(P)$. A more general concept was introduced in [9, Def. 2.8] for arbitrary simplicial complexes, but it was not given a name there. In their notation, we have ${{\,\mathrm{Tr}\,}}_i(P) = {\mathcal {F}}_P^{(i,d)}$.

Example 2.2 shows that the 2-trunk of a 2-dimensional tropical polytope in 4-dimensional space is not necessarily connected.

#### Example 2.2

**Fig. 2:**
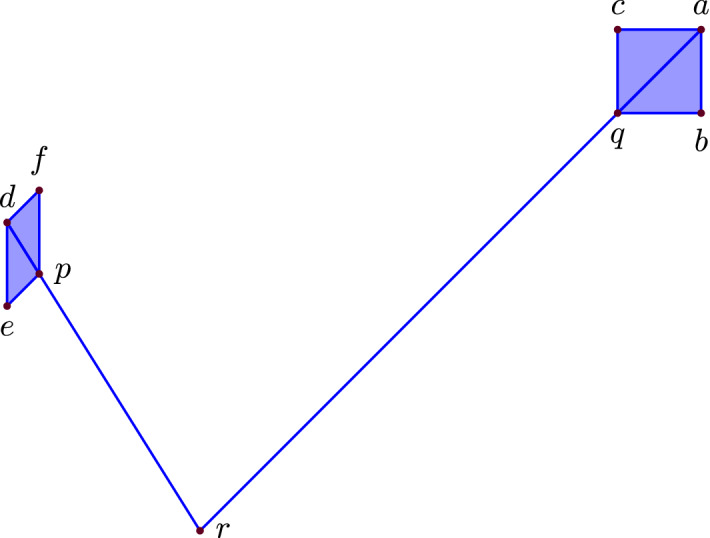
A 4-dimensional tropical polytope whose 2-trunk is disconnected

The tropical polytope spanned by the following points:is visualized in Fig. 2. All pseudovertices are marked in purple, we have the additional pseudoverticesThe maximal cells of the corresponding covector decomposition, computed with polymake [23], are $\{rp,rq, ped, pfd, qba, qca\}.$

A particularly nice class of tropical polytopes are the *pure tropical polytopes*, that is, those which coincide with their *d*-trunk. The well-behaved nature of pure tropical polytopes was used to exhibit canonical exterior descriptions in [4]. In a similar spirit, the following statement uses a technique already occurring in the study of minimal external representations of tropical polytopes [3]. In contrast to the disconnectedness of the 2-trunk in Example 2.2, it shows in particular that the *d*-trunk of a tropical polytope in ${\mathbb {T}}^d$ is a tropical polytope itself.

#### Proposition 2.3

The tropical convex hull of two full-dimensional pure tropical polytopes is a pure, full-dimensional tropical polytope.

Consequently, the *d*-trunk of a tropical polytope in ${\mathbb {T}}^d$ is a tropical polytope.

#### Proof

Let *P* and *Q* be two full-dimensional pure tropical polytopes in ${\mathbb {T}}^d$ and let $\mathring{P}$ and $\mathring{Q}$ be their interior. Clearly, we have ${{\,\mathrm{tconv}\,}}(P \cup Q) \supseteq \overline{{{\,\mathrm{tconv}\,}}(\mathring{P} \cup \mathring{Q})}$, where ${\overline{S}}$ denotes the closure in the usual topology of a set *S*. As *P* and *Q* are pure, we have $\overline{\mathring{P}} = P$ and $\overline{\mathring{Q}} = Q$. Let $t = \bigoplus _{r \in R} \lambda _r \odot r \oplus \bigoplus _{s \in S} \lambda _s \odot s$ for some finite subsets $R \subset P, S \subset Q$ be a point in ${{\,\mathrm{tconv}\,}}(P \cup Q)$ and let $(r_i)_{i \in {\mathbb {N}}} \rightarrow r$ for each $r \in R$ and $(s_i)_{i \in {\mathbb {N}}} \rightarrow s$ for each $s \in S$ be sequences in $\mathring{P}$ and $\mathring{Q}$, respectively. By the continuity of the operations $\max$ and ‘$+$’ we obtain

$$\begin{aligned} \left( \bigoplus _{r \in R} \lambda _r \odot r_i \oplus \bigoplus _{s \in S} \lambda _s \odot s_i\right) _{i \in {\mathbb {N}}} \rightarrow t . \end{aligned}$$

Together with the other inclusion, this shows ${{\,\mathrm{tconv}\,}}(P \cup Q) = \overline{{{\,\mathrm{tconv}\,}}(\mathring{P} \cup \mathring{Q})}$. For $\varepsilon > 0$, we define

$$\begin{aligned} B_{\varepsilon } = {{\,\mathrm{tconv}\,}}\begin{pmatrix} -\varepsilon &{}\quad \varepsilon &{}\quad 0 &{}\quad \cdots &{}\quad 0 \\ -\varepsilon &{}\quad 0 &{}\quad \varepsilon &{}\quad \cdots &{}\quad 0 \\ \vdots &{}\quad 0 &{}\quad \ddots &{}\quad \ddots &{}\quad \vdots \\ - \varepsilon &{}\quad 0 &{} \quad \cdots &{}\quad 0 &{}\quad \varepsilon \end{pmatrix} , \end{aligned}$$

a full-dimensional polytrope.

For any two points $p \in \mathring{P}$ and $q \in \mathring{Q}$ there is a sufficiently small $\varepsilon > 0$ such that $p + B_{\varepsilon } \subseteq \mathring{P}$ and $q + B_{\varepsilon } \subseteq \mathring{Q}$. Then the ‘inflated tropical line’ ${{\,\mathrm{tconv}\,}}(p,q) + B_{\varepsilon }$ is contained in ${{\,\mathrm{tconv}\,}}(\mathring{P} \cup \mathring{Q})$. Therefore, each point is surrounded by a small full-dimensional polytrope in ${{\,\mathrm{tconv}\,}}(\mathring{P} \cup \mathring{Q})$. This implies that each point of $\overline{{{\,\mathrm{tconv}\,}}(\mathring{P} \cup \mathring{Q})}$ is in the closure of a full-dimensional cell. Hence, ${{\,\mathrm{tconv}\,}}(P \cup Q) = \overline{{{\,\mathrm{tconv}\,}}(\mathring{P} \cup \mathring{Q})}$ is pure and full-dimensional.

The polytropes in the covector decomposition of the *d*-trunk are full-dimensional pure tropical polytopes *P*. Hence, the tropical convex hull of their union is a full-dimensional pure tropical polytope. Moreover, it is contained in the *d*-trunk of *P*, as it is a subset of *P*. Therefore, the tropical convex hull of the *d*-trunk of *P* is just the *d*-trunk itself. $\square$

The covector decomposition of a tropical polytope $P = {{\,\mathrm{tconv}\,}}(V)$, where *V* has only integral entries, is formed of alcoved polytopes in the sense of Lam and Postnikov [32]. They studied triangulations and lattice points of alcoved polytopes from a classical point of view, while we are heading toward tropical metric estimates. Each such alcoved polytope has a triangulation into simplices of the form

$$\begin{aligned} \Delta _\pi (a) := {{\,\mathrm{conv}\,}}\left\{ a + e_{\pi (1)} + \cdots + e_{\pi (\ell )} : \ell =0,1,\ldots ,d \right\} , \end{aligned}$$

where $\pi \in S_d$ is a permutation of the coordinates and $a \in {\mathbb {Z}}^d$. For $\pi = id$, we just write $\Delta (a) := \Delta _{id}(a)$. We denote the simplicial complex formed by these *alcoved simplices* by ${\mathcal {T}}_P$ and call it the *alcoved triangulation* of *P*. The inequality description of $\Delta ({\mathbf {0}})$ is given by

$$\begin{aligned} \Delta ({\mathbf {0}}) = \left\{ x \in {\mathbb {R}}^d : 0 \le x_d \le x_{d-1} \le \cdots \le x_1 \le 1 \right\} \end{aligned}$$

(cf. [7, Ch. 7]), where the all-zeroes vector is denoted by ${\mathbf {0}}= (0,\ldots ,0)^\intercal$. We use the following notation to compactly index (half-)open faces of $\Delta ({\mathbf {0}})$: For $s = (\prec _1,\prec _2,\ldots ,\prec _{d+1}) \in \left\{ =,\le ,<\right\} ^{d+1}$, we write

$$\begin{aligned} \Delta ^s({\mathbf {0}}) = \left\{ x \in {\mathbb {R}}^d : 0 \prec _{d+1} x_d \prec _d x_{d-1} \prec _{d-1} \cdots \prec _2 x_1 \prec _1 1 \right\} , \end{aligned}$$

and

$$\begin{aligned} \Delta ^s(a) = a + \Delta ^s({\mathbf {0}}) . \end{aligned}$$

### Tropical lattices

Recent advances on the complexity of linear programming using tropical geometry [2] demonstrated a fruitful use of metric estimates for tropical polyhedra. In classical convex geometry, the number of lattice points can be interpreted as a discrete version of a volume. This raises the question what ‘tropical integers’ or ‘tropical natural numbers’ should be.

The nonnegative integers form a submonoid of the additive monoid $({\mathbb {R}},+)$ generated by 1. The analogous tropical construction does not lead to a rich structure, as tropical addition is idempotent, and so $0 \oplus 0 = 0$.

Another approach comes from the property of lattices to be spanned by a finite discrete set. In particular, as lattices correspond to discrete additive subgroups of ${\mathbb {R}}^d$, they have a fix-group of translations. Although this perspective has been used in [22] and allows a tropical Ehrhart theory connected to the Euclidean volume of the polytopes in the covector decomposition (see Sect. 3), it is too rough for our purposes.

Instead, we propose to consider the set $\Gamma _b := \log _b({\mathbb {Z}}_{\ge 0})$ as a concept for tropical integers, where $b \ge 2$ is an arbitrarily chosen natural number. This is natural in the sense that it respects the operation-wise transition from $({\mathbb {R}},+,\cdot )$ to the tropical semiring $({\mathbb {R}}\cup \{-\infty \},\max ,+)$:

$$\begin{aligned} ({\mathbb {Z}},+,\cdot ) \quad \longrightarrow \quad ({\mathbb {Z}}_{\ge 0},\max ,\cdot ) \longrightarrow (\log _b({\mathbb {Z}}_{\ge 0}),\max ,+). \end{aligned}$$

As additional motivation, the set $\Gamma _b$ satisfies a tropicalization of the identity

$$\begin{aligned} \#\,\left( [0,k \cdot v) \cap {\mathbb {Z}}_{\ge 0}\right) = k \cdot \#\,\left( [0,v) \cap {\mathbb {Z}}_{\ge 0}\right) \quad \text {for}\quad k,v \in {\mathbb {Z}}_{\ge 0} . \end{aligned}$$

Indeed, we have

$$\begin{aligned} \#\,\left( [-\infty ,k \odot v) \cap \Gamma _b\right) = b^k \cdot \#\,\left( [-\infty ,v) \cap \Gamma _b\right) \quad \text {for} \; k \in {\mathbb {Z}}_{\ge 0} \text { and } v \in \Gamma _b . \end{aligned}$$

Our main concept of tropical lattice is therefore the following. It grasps the aspect of the lattice ${\mathbb {Z}}^d$ in classical Ehrhart theory that the number of lattice points contained in a polytope suitably increases by dilation.

#### Definition 2.4

(*Tropical* *b*-*lattice*) Define the *tropical* *b*-*lattice* in ${\mathbb {T}}^d$ by

$$\begin{aligned} \Gamma _b^d:= \left( \log _b({\mathbb {Z}}_{\ge 0})\right) ^d = \left\{ \left( \log _b(x_1),\ldots ,\log _b(x_d)\right) ^\intercal : x_1,\ldots ,x_d \in {\mathbb {Z}}_{\ge 0} \right\} . \end{aligned}$$

As we often want to vary *b*, we define the *tropical canonical lattice* ${\mathbb {T}}{\mathbb {N}}^d := ({\mathbb {Z}}_{\ge 0} \cup \{-\infty \})^d$. Tropical *b*-lattices intersect exactly in ${\mathbb {T}}{\mathbb {N}}^d$:

#### Lemma 2.5

$$\begin{aligned} {\mathbb {T}}{\mathbb {N}}^d = \bigcap _{b \in {\mathbb {N}}_{\ge 2}} \log _b({\mathbb {Z}}_{\ge 0})^d . \end{aligned}$$

#### Proof

It suffices to prove the identity for $d=1$. One inclusion is straightforward. In fact, for every $m \in {\mathbb {T}}{\mathbb {N}}= {\mathbb {Z}}_{\ge 0} \cup \{-\infty \}$ and every $b \in {\mathbb {N}}_{\ge 2}$ we have $m = \log _b(b^m)$, with the convention that $b^{-\infty }=0$.

For the reverse inclusion, we first argue that any non-integral number in the intersection $\bigcap _{b \in {\mathbb {N}}_{\ge 2}} \log _b({\mathbb {Z}}_{\ge 0})$ has to be transcendental. To this end, for any $b \in {\mathbb {N}}_{\ge 2}$, by the Gelfond–Schneider Theorem (cf. [36, § 2.1]) $b^x$ is transcendental whenever *x* is algebraic over ${\mathbb {Z}}$ and irrational. Therefore, every $x \in \log _b({\mathbb {Z}}_{\ge 0})$ is either rational or transcendental. Assume that $x=p/q$ is rational. We get an integer $m \in {\mathbb {N}}$ such that $p/q = \log _b(m)$, or equivalently, $m^q = b^p$. Thus, for prime *b*, we must have *q* dividing *p*, and thus $x=p/q$ is an integer.

Now, if $x \in \bigcap _{b \in {\mathbb {N}}_{\ge 2}} \log _b({\mathbb {Z}}_{\ge 0})$ would be transcendental, then $x, x^2, x^3$ are linearly independent over ${\mathbb {Q}}$. Furthermore, $\ln (2)$ and $\ln (3)$ are linearly independent over ${\mathbb {Q}}$ as well, otherwise an integral power of 2 would coincide with an integral power of 3, a contradiction. Apply now the Six Exponentials Theorem (cf. [36, § 3.2]) to $x_i=x^i$, for $i=1,2,3$, and $y_1=\ln (2)$, $y_2=\ln (3)$. As a result, at least one of the numbers $e^{x_iy_j}$ is transcendental. However, these exponentials are equal either to $2^{x^i}$ or $3^{x^i}$, which were all assumed integers. For example, $2^{x^2} = (2^x)^x$ is an integer using the base $b=2^x$. This contradiction shows that any $x \in \bigcap _{b \in {\mathbb {N}}_{\ge 2}} \log _b({\mathbb {Z}}_{\ge 0})$ must be an integer. $\square$

#### Definition 2.6

(*Tropical lattice polytopes*) Let $b \in {\mathbb {N}}_{\ge 2}$. A tropical polytope whose vertices all lie in $\Gamma _b^d$ is called a *tropical* *b*-*lattice polytope*. If also all pseudovertices lie in $\Gamma _b^d$, then we call it a *strong tropical* *b*-*lattice polytope*. *Tropical (canonical) lattice polytopes* are those whose vertices lie in ${\mathbb {T}}{\mathbb {N}}^d$.

Tropical canonical lattice polytopes were already studied with a different motivation by Zhang [38]. They are compatible with the covector decomposition in the sense that the pseudovertices belong to ${\mathbb {T}}{\mathbb {N}}^d$ and to $\Gamma _b^d$, for every $b \in {\mathbb {N}}_{\ge 2}$. This is however not true for (non-strong) tropical *b*-lattice polytopes in general, as demonstrated by the tropical 5-lattice polytope with vertices

$$\begin{aligned} (0,0)^{\intercal },\quad (\log _5 3,\log _5 2)^{\intercal },\quad (\log _5 2,\log _5 4)^{\intercal } . \end{aligned}$$

### Different versions of convexity

Tropical convexity is mainly associated with the semiring $S_{(\max ,+)} = ({\mathbb {R}}\cup \{-\infty \}, \max , +)$ or, by applying the semiring isomorphism $x \mapsto -x$, the semiring $({\mathbb {R}}\cup \{\infty \}, \min , +)$. In the notation introduced before, we have ${\mathbb {T}}= S_{(\max ,+)}$. We use the latter notation whenever we need to emphasize the different semirings, and we employ the shorter and more common notation ${\mathbb {T}}$ otherwise.

While transferring from $S_{(\max ,+)}$ to $S_{(\max ,\cdot )} = ({\mathbb {R}}_{> 0} \cup \{0\},\max ,\cdot )$ via the semiring isomorphism $\exp _b :x \mapsto b^x$ is often merely a structural reformulation, it has a benefit for our metric considerations, because it relates the lattice point structures over $S_{(\max ,+)}$ and $S_{(\max ,\cdot )}$. The next claim is far from true for general polytopes but due to the special structure of polytropes.

#### Proposition 2.7

The image under the map $\exp _b$ of a polytrope is a polytope.

#### Proof

The defining inequalities of a polytrope are of the form $c \le x_i$, $c \ge x_i$, or $x_i \le x_j + c$, for $i \ne j$, see [27]. As

$$\begin{aligned} \exp _b\left( \{x \in {\mathbb {R}}^d :c \ge x_i \}\right) = \{x \in {\mathbb {R}}^d :\exp _b(c) \ge x_i \} , \end{aligned}$$

and analogously with $\le$ instead of $\ge$, as well as,

$$\begin{aligned} \exp _b\left( \{x \in {\mathbb {R}}^d :x_i \le x_j + c \}\right) = \{x \in {\mathbb {R}}^d :x_i \le \exp _b(c) \cdot x_j \} , \end{aligned}$$

the statement follows by taking the intersection of such sets. $\square$

More generally, the image of a *weighted digraph polyhedron* [28] under the exponentiation map results in a particular *distributive polyhedron* as studied in [19].

Consider a semiring *S* with addition $\oplus _S$ and multiplication $\odot _S$ with neutral elements $0_S$ and $1_S$, respectively. A polytope over *S* is the set of finite combinations $\lambda _1 \odot _S v_1 \oplus _S \dots \oplus _S \lambda _n \odot _S v_n$ of elements $v_1,\ldots ,v_n \in V$ of a finite set $V \subset S^d$ with coefficients $\lambda _1,\ldots ,\lambda _n \in S$ which sum up to $1_S$.

While a *polytope over* $S_{(\max ,+)}$ is a just a tropical polytope as defined in (2), its image under a semiring isomorphism $\exp _b$, for some $b \in {\mathbb {R}}_{\ge 0}$, is a *polytope over* $S_{(\max ,\cdot )}$. Proposition 2.7 shows that we obtain a polyhedral complex subdividing a polytope *P* over $S_{(\max ,\cdot )}$, as the image of the covector decomposition of the polytope $\log _b(P)$ over $S_{(\max ,+)}$. We call this again the covector decomposition and its vertices the pseudovertices.

**Fig. 3:**
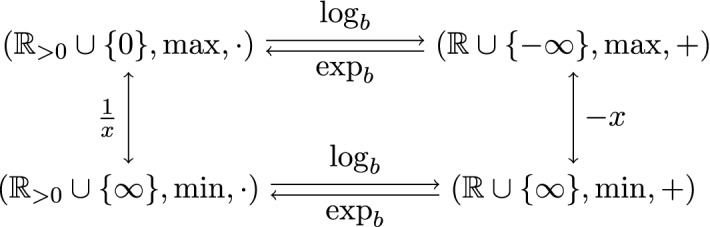
Commutative diagram of several semiring isomorphisms

A summary of the semiring isomorphisms and other involved maps is shown in Fig. 3.

## Tropical Ehrhart polynomials

### Lattice point counting and semiring isomorphisms

Ehrhart’s theorem on the polynomiality of the counting function $k \mapsto L(P,k)$ of a lattice polytope $P \subseteq {\mathbb {R}}^d$, has the following powerful extension to complexes of lattice polytopes.

#### Theorem 3.1

([7, Cor. 5.6.1]) Let ${\mathcal {K}}$ be a complex of lattice polytopes in ${\mathbb {R}}^d$ and let $|{\mathcal {K}} | = \bigcup _{P \in {\mathcal {K}}} P$ be its underlying point set. Then, the counting function $k \mapsto \#\,\left( k|{\mathcal {K}} | \cap {\mathbb {Z}}^d\right)$ agrees with a polynomial of degree $\dim ({\mathcal {K}})$ for all positive integers $k \in {\mathbb {N}}$.

We saw in Sect. 2.3 that a polytope over $S_{(\max ,\cdot )}$ has a natural structure as a polyhedral complex. The appropriate lattice for the semiring $S_{(\max ,\cdot )}$ is ${\mathbb {Z}}^d_{\ge 0}$ and thus consists of integral vectors. In analogy with classical lattice polytopes in ${\mathbb {R}}^d$, we thus call a polytope over $S_{(\max ,\cdot )}$ a *lattice polytope* if all its vertices are *lattice points*, meaning that they belong to ${\mathbb {Z}}^d_{\ge 0}$. We call such a polytope a *strong lattice polytope* if *all* pseudovertices of its covector decomposition are contained in ${\mathbb {Z}}^d_{\ge 0}$. Via the isomorphism between $S_{(\max ,\cdot )}$ and $S_{(\max ,+)}$ these notions correspond to those in Definition 2.6. We need to make this distinction for the sake of applicability of Theorem 3.1. Indeed, we get the following:

#### Theorem 3.2

For a strong lattice polytope $P \subseteq (S_{(\max ,\cdot )})^d$, the counting function $k \mapsto \#\,\left( kP \cap {\mathbb {Z}}^d_{\ge 0}\right)$ agrees with a polynomial of degree $\dim (P) \le d$ for all positive integers $k \in {\mathbb {N}}$.

The coefficient of $k^d$ equals the Euclidean volume of *P*.

**Fig. 4:**
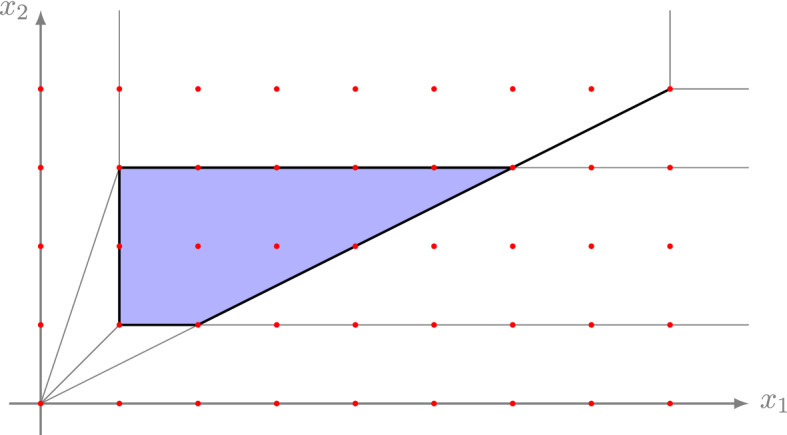
The shaded area forms a strong lattice polytope over $S_{(\max ,\cdot )}$

A natural question that arises is

#### Question 3.3

How can we tell from the vertices if they span a strong lattice polytope over $S_{(\max ,\cdot )}$?

Going back to tropical canonical lattice polytopes (see Definition 2.6), we actually obtain two different polynomials; one counting the lattice points in ${\mathbb {Z}}^d$, the other one counting *b*-lattice points. The first version is less natural from the semiring operations, but it was used in [22].

#### Theorem 3.4

For a tropical lattice polytope $P \subseteq (S_{(\max ,+)})^d = {\mathbb {T}}^d$ the counting function $k \mapsto \#\,\left( k\cdot P \cap {\mathbb {Z}}^d\right)$ agrees with a polynomial of degree $\dim (P)$ for all positive integers $k \in {\mathbb {N}}$.

**Fig. 5:**
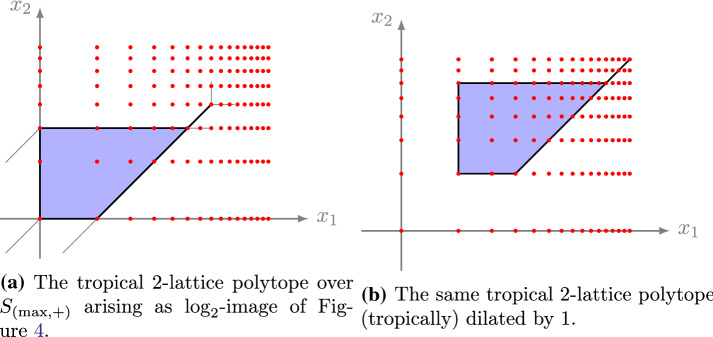
The lattice points are condensed by $\log _2$ such that the tropical dilation implies a polynomial increase in the number of contained lattice points

The next concept is at the heart of our quantitative studies.

#### Definition 3.5

Let $P \subseteq {\mathbb {T}}^d$ be a tropical lattice polytope and let $b \in {\mathbb {N}}_{\ge 2}$. We define the *tropical lattice point enumerator* of *P* (with respect to *b*) as

$$\begin{aligned} {\mathfrak {L}}_P^b(k) := \#\,\left( (k \odot P) \cap \Gamma _b^d\right) , \quad k \in {\mathbb {Z}}_{\ge 0} . \end{aligned}$$

Applying the semiring isomorphism $\log _b$ to Theorem 3.2 we obtain

#### Theorem 3.6

(Tropical Ehrhart polynomial) Let $b \in {\mathbb {N}}_{\ge 2}$ and let $P \subseteq {\mathbb {T}}^d$ be a tropical lattice polytope. The tropical lattice point enumerator ${\mathfrak {L}}_P^b(k)$ agrees with a polynomial in $b^k$ for every $k \in {\mathbb {Z}}_{\ge 0}$.

#### Proof

The set $Q = \exp _b(P)$ is a strong lattice polytope over $S_{(\max ,\cdot )}$. Hence, by Theorem 3.2, there is a polynomial *q* of degree $\dim (Q) = \dim (P)$ with

$$\begin{aligned} q(\ell ) = \#\,\left( \ell Q \cap {\mathbb {Z}}^d\right) \end{aligned}$$

for all $\ell \in {\mathbb {Z}}_{>0}$. Substituting $b^k$ for $\ell$ and using $Q = \exp _b(P) \subseteq {\mathbb {R}}_{\ge 0}^d$, we get

$$\begin{aligned} q(b^k) = \#\,\left( \log _b\left( b^k \cdot \exp _b(P) \cap {\mathbb {Z}}_{\ge 0}^d \right) \right) = \#\,\left( (k \odot P) \cap \Gamma _b^d\right) . \end{aligned}$$

Note the use of the semiring homomorphism property of $\log _b$. $\square$

#### Remark 3.7

The proof above shows that the Ehrhart polynomials of $P={{\,\mathrm{tconv}\,}}(M) \subseteq (S_{(\max ,+)})^d$ and $Q=\exp _b(P) \subseteq (S_{(\max ,\cdot )})^d$ agree up to a change of variables. More precisely, we have ${\mathfrak {L}}_P^b(k) = q(b^k)$, for all $k \in {\mathbb {Z}}_{\ge 0}$.

#### Remark 3.8

If one relaxes the integrality assumption in the classical setting and considers *rational* polytopes $P \subseteq {\mathbb {R}}^d$, that is, polytopes all of whose vertices have only rational coordinates, then their Ehrhart function $k \mapsto \#\,\left( kP \cap {\mathbb {Z}}^d\right)$ turns out to be a quasi-polynomial (cf. [6, Ch. 3.8]).

In the various scenarios discussed above, rationality may be defined as follows:

- a polytope over $S_{(\max ,\cdot )}$ is *rational* if all its pseudovertices are rational,
- a polytope over $S_{(\max ,+)}$ is *tropically rational* if all its pseudovertices are integral (allowing possibly negative coordinates),
- a tropical polytope $P \subseteq {\mathbb {T}}^d$ is *tropically* *b*-*rational* if all its pseudovertices are contained in $\log _b({\mathbb {Q}}_{\ge 0})^d$.

The methods that we employed above to prove polynomiality, can similarly be used to show that in all three cases above the corresponding Ehrhart functions are quasi-polynomials as well.

#### Definition 3.9

(*Tropical Ehrhart coefficients*) Let $P \subseteq {\mathbb {T}}^d$ be a tropical lattice polytope. We write

$$\begin{aligned} {\mathfrak {L}}_P^b(k) = \sum _{i=0}^d c_i^b(P) (b^k)^i \end{aligned}$$

for its tropical Ehrhart polynomial and we call $c_i^b(P)$ the *i*th *tropical Ehrhart coefficient* of *P*.

A very useful and reoccurring phenomenon in geometric combinatorics is *reciprocity* (see [7] for a detailed account). For lattice point counting functions this is known as Ehrhart–MacDonald reciprocity (cf. [6, Ch. 4]) and refers to the fact that evaluating the Ehrhart polynomial $L(P,k) = \sum _{i=0}^d c_i(P) k^i$ of a lattice polytope $P \subseteq {\mathbb {R}}^d$ at negative integers amounts to counting lattice points in the $k^{th}$ dilate of the interior $\mathop {P}\limits ^{\circ }$ of *P*. That is,

$$\begin{aligned} L(P,-k)&= (-1)^{\dim (P)} L(\mathop {P}\limits ^{\circ },k)\quad \text {for}\quad k \in {\mathbb {Z}}_{>0}. \end{aligned}$$

We say that a counting function satisfying this relation *fulfills reciprocity*.

If a lattice polytope over $S_{(\max ,\cdot )}$ is pure, defined analogously for polytopes over $S_{(\max ,\cdot )}$ as over $S_{(\max ,+)}$, the polyhedral complex induced by its covector decomposition is a *d*-manifold and by [33] reciprocity holds.

#### Theorem 3.10

(i) The Ehrhart polynomial in Theorem 3.2 of a pure strong lattice polytope over $S_{(\max ,\cdot )}$ and the Ehrhart polynomial in Theorem 3.4 of a pure tropical lattice polytope over $S_{(\max ,+)}$ fulfill reciprocity.
(ii) The tropical Ehrhart polynomial ${\mathfrak {L}}_P^b(k)$ of a pure tropical lattice polytope $P \subseteq {\mathbb {T}}^d$ satisfies a reciprocity law in the sense that
  $$\begin{aligned} c_i^b(\mathop {P}\limits ^{\circ }) = (-1)^{d-i} c_i^b(P)\quad \text {for}\quad i \in \{0,1,\ldots ,d\}. \end{aligned}$$

### Explicit expressions for tropical Ehrhart coefficients

In this section, we take a much more refined route to Theorem 3.6 which is based on combining the covector decomposition with tools from classical Ehrhart theory. This allows for a refined representation of the tropical Ehrhart coefficients and leads to our desired tropical volume concept. For comparison to ordinary Ehrhart theory and further reading, we refer to [6].

In order to formulate our main technical lemma, we denote the diagonal matrix with diagonal entries $b^{a_i}$ by $D_b^a = {{\,\mathrm{diag}\,}}(b^{a_1},\ldots ,b^{a_d}) \in {\mathbb {Z}}^{d \times d}$, for $a \in {\mathbb {Z}}_{\ge 0}^d$ and $b \in {\mathbb {N}}_{\ge 2}$. Further, let $s = (\prec _1,\prec _2,\ldots ,\prec _{d+1}) \in \left\{ =,\le ,<\right\} ^{d+1}$. We denote the all-one vector by ${\mathbf {1}}= (1,\ldots ,1)^\intercal$.

#### Lemma 3.11

For every $k \in {\mathbb {Z}}_{\ge 0}$, the map $\phi : {\mathbb {R}}_{>0}^d \rightarrow {\mathbb {R}}^d$ defined by $\phi (z) = (\log _b(z_1),\dots ,\log _b(z_d))^\intercal$ induces a bijection between

$$\begin{aligned} \left( b^k D_b^a {\mathbf {1}}+ (b^{k+1}-b^k) D_b^a \Delta ^s({\mathbf {0}}) \right) \cap {\mathbb {Z}}_{\ge 0}^d \quad \text {and}\quad \left( k \odot \Delta ^s(a) \right) \cap \Gamma _b^d. \end{aligned}$$

#### Proof

Clearly, $\phi$ is bijective and by definition it maps points in ${\mathbb {Z}}_{\ge 0}^d$ to points in $\Gamma _b^d= (\log _b({\mathbb {Z}}_{\ge 0}))^d$. So what we need to check is that $z \in b^k D_b^a {\mathbf {1}}+ (b^{k+1}-b^k) D_b^a \Delta ^s({\mathbf {0}})$ if and only if $\phi (z) \in \left( k \odot \Delta ^s(a) \right) = k {\mathbf {1}}+ a + \Delta ^s({\mathbf {0}})$. As we saw above, the inequality description of the simplex $\Delta ^s({\mathbf {0}})$ is given by

$$\begin{aligned} \Delta ^s({\mathbf {0}}) = \left\{ x \in {\mathbb {R}}^d : 0 \prec _{d+1} x_d \prec _d x_{d-1} \prec _{d-1} \cdots \prec _2 x_1 \prec _1 1 \right\} . \end{aligned}$$

Therefore, $z \in b^k D_b^a {\mathbf {1}}+ (b^{k+1}-b^k) D_b^a \Delta ^s({\mathbf {0}})$ if and only if

$$\begin{aligned}&0 \prec _{d+1} \frac{z_d}{b^{a_d}} - b^k \prec _d \frac{z_{d-1}}{b^{a_{d-1}}} - b^k \prec _{d-1} \cdots \prec _2 \frac{z_1}{b^{a_1}} - b^k \prec _1 b^{k+1}-b^k \\&\quad \Longleftrightarrow b^k \prec _{d+1} \frac{z_d}{b^{a_d}} \prec _d \frac{z_{d-1}}{b^{a_{d-1}}} \prec _{d-1} \cdots \prec _2 \frac{z_1}{b^{a_1}} \prec _1 b^{k+1} \\&\quad \Longleftrightarrow k \prec _{d+1} \log _b(z_d) - a_d \prec _d \cdots \prec _2 \log _b(z_1) - a_1 \prec _1 k+1, \end{aligned}$$

which holds if and only if $\phi (z) = (\log _b(z_1),\ldots ,\log _b(z_d))^\intercal \in k {\mathbf {1}}+ a + \Delta ^s({\mathbf {0}})$. Here we also used that the logarithm $x \mapsto \log _b(x)$ is strictly increasing. $\square$

#### Example 3.12

The alcoved simplex $${{\,\mathrm{conv}\,}}\begin{pmatrix} 3 &{} 4 &{} 4 \\ 5 &{} 5 &{} 6 \end{pmatrix} = 2 \odot {{\,\mathrm{conv}\,}}\begin{pmatrix} 1 &{} 2 &{} 2 \\ 3 &{} 3 &{} 4 \end{pmatrix}$$ maps to $$7^2 \cdot {{\,\mathrm{conv}\,}}\begin{pmatrix} 7^1 &{} 7^2 &{} 7^2 \\ 7^3 &{} 7^3 &{} 7^4 \end{pmatrix} = 7^2 \cdot \left( \begin{pmatrix} 7^1 \\ 7^3 \end{pmatrix} + {{\,\mathrm{conv}\,}}\begin{pmatrix} 0 &{} 6\cdot 7 &{} 6 \cdot 7 \\ 0 &{} 0 &{} 6\cdot 7^3 \end{pmatrix} \right) = 7^2 \cdot \left( D_{7}^{(1,3)} {\mathbf {1}}+ 6 \cdot D_{7}^{(1,3)} \cdot {{\,\mathrm{conv}\,}}\begin{pmatrix} 0 &{} 1 &{} 1 \\ 0 &{} 0 &{} 1 \end{pmatrix} \right) $$ via $\exp _7$.

The proof of Lemma 3.11 suggests that the tropical Ehrhart polynomial is close to a weighted version of the usual Ehrhart polynomial with weight function $z \mapsto b^z$. Weighted Ehrhart polynomials have been studied, for instance, by Baldoni et al. [5] (they use polynomial weight functions but also discuss exponential weights).

#### Example 3.13

The Ehrhart polynomial of a lattice polygon $P \subseteq {\mathbb {R}}^2$ equals

$$\begin{aligned} \#\,\left( kP \cap {\mathbb {Z}}^2 \right) = {{\,\mathrm{vol}\,}}(P) k^2 + \tfrac{1}{2} \#\,\left( \partial P \cap {\mathbb {Z}}^2 \right) k + 1. \end{aligned}$$

Since ${{\,\mathrm{vol}\,}}(D_b^a \Delta ({\mathbf {0}})) = \tfrac{1}{2} b^{a_1+a_2}$ and $\#\,\left( \partial D_b^a \Delta ({\mathbf {0}}) \cap {\mathbb {Z}}^2\right) = b^{a_1} + b^{a_2} + b^{\min (a_1,a_2)}$, we use Lemma 3.11 and we get the tropical Ehrhart polynomial of $\Delta (a)$, for each $a \in {\mathbb {Z}}_{\ge 0}^2$:

$$\begin{aligned} {\mathfrak {L}}_{\Delta (a)}^b(k)&= \tfrac{1}{2} b^{a_1+a_2} (b^{k+1} - b^k)^2 + \tfrac{1}{2} (b^{a_1} + b^{a_2} + b^{\min (a_1,a_2)}) (b^{k+1} - b^k) + 1\\&= \tfrac{1}{2} (b-1)^2 b^{a_1+a_2} (b^k)^2 + \tfrac{1}{2} (b-1) (b^{a_1} + b^{a_2} + b^{\min (a_1,a_2)}) b^k + 1. \end{aligned}$$

The following is our desired precise version of Theorem 3.6, building on the structure of the covector decomposition discussed in Sect. 2.1. In particular, we use the alcoved triangulation ${\mathcal {T}}_P$. It expresses the tropical Ehrhart coefficients as signed and weighted sums of the classical Ehrhart coefficients of diagonally transformed alcoved simplices.

#### Theorem 3.14

The *i*th tropical Ehrhart coefficient of the tropical lattice polytope $P \subseteq {\mathbb {T}}^d$ is given by3where ${\overline{Q}}$ denotes the closure of a set $Q \subseteq {\mathbb {R}}^d$.

#### Proof

Every element of the alcoved triangulation ${\mathcal {T}}_P$ of *P*, as discussed in Sect. 2.1, is of the form $\Delta _\pi ^s(a)$, for some $s \in \{=,\le ,<\}^{d+1}$ and $a \in {\mathbb {Z}}_{\ge 0}$. Moreover, we think of these alcoved simplices as being relatively open, that is, $s \in \{=,<\}^{d+1}$, since this yields a partition of *P* into these pieces.

Therefore, the tropical lattice point enumerator ${\mathfrak {L}}_P^b(k) = \#\,\left( (k \odot P) \cap \Gamma _b^d\right)$ is the sum of the functions ${\mathfrak {L}}_{\Delta ^s_\pi (a)}^b(k)$. By Lemma 3.11, we have

$$\begin{aligned} {\mathfrak {L}}_{\Delta ^s_\pi (a)}^b(k) = \#\,\left( \left( k \odot \Delta ^s_\pi (a) \right) \cap \Gamma _b^d\right) = \#\,\left( \left( (b^{k+1}-b^k) D_b^a \Delta ^s_\pi ({\mathbf {0}}) \right) \cap {\mathbb {Z}}^d \right) . \end{aligned}$$

Now, $D_b^a \Delta ^s_\pi ({\mathbf {0}})$ is a relatively open simplex all of whose vertices lie in ${\mathbb {Z}}^d$ and whose dimension is

$$\begin{aligned} m = \#\{i : s_i = \text {`} < \text {'}\} - 1. \end{aligned}$$

Classical Ehrhart Theory on the standard lattice ${\mathbb {Z}}^d$ (cf. [6, Ch. 3]) implies that ${\mathfrak {L}}_{\Delta ^s_\pi (a)}^b(k)$ agrees with a polynomial in $b^{k+1}-b^k$ of degree *m*, whose coefficients depend on $\pi , a, s$, and *b*, but not on *k*. Thus, ${\mathfrak {L}}_{\Delta ^s_\pi (a)}^b(k)$ agrees with a polynomial in $b^k$ for every $k \in {\mathbb {Z}}_{\ge 0}$. We conclude by observing that ${\mathfrak {L}}_P^b(k)$ as a sum of polynomials, is a polynomial in $b^k$ as well.

In order to derive the stated formula for the *i*th tropical Ehrhart coefficient of *P*, we writeBy Ehrhart reciprocity [6, Thm. 4.1] we getSubstituting $t = (b-1)b^k$ and summing over all at least *i*-dimensional elements in ${\mathcal {T}}_P$ as described above finishes the proof. $\square$

#### Corollary 3.15

The highest nonvanishing tropical Ehrhart coefficient of a tropical lattice polytope $P \subseteq {\mathbb {T}}^d$ is indexed by the dimension of ${\mathcal {T}}_P$.

#### Proof

Let *k* be the dimension of ${\mathcal {T}}_P$. As for the classical Ehrhart polynomial, the highest nonvanishing coefficient is indexed by the dimension of the polytope, the right-hand side of (3) shows that $c_i^b(P) = 0$, for every $i > k$. Moreover, for $i=k$ the expression (3) reduces towhich is nonzero, as the sum is non-empty and every summand is positive (the highest nonvanishing Ehrhart coefficient equals the relative volume of the considered polytope; cf. [6, Sect. 5.4]). $\square$

### First properties of tropical Ehrhart coefficients

Here, we record two properties of tropical Ehrhart coefficients that go well in line with their classical counterparts. We write ${\mathcal {P}}_{{\mathbb {T}},{\mathcal {L}}}^d$ for the family of tropical lattice polytopes in ${\mathbb {T}}^d$.

#### Proposition 3.16

Let $P \in {\mathcal {P}}_{{\mathbb {T}},{\mathcal {L}}}^d$ and let $i \in \{0,1,\ldots ,d\}$.

(i) *(Homogeneity)* For every $\lambda \in {\mathbb {Z}}_{\ge 0}$, we have
  $$\begin{aligned} c_i^b(\lambda \odot P) = (b^\lambda )^i \cdot c_i^b(P) . \end{aligned}$$
(ii) *(Valuation property)* For every $b \in {\mathbb {N}}_{\ge 2}$, the function $c_i^b(\cdot ) : {\mathcal {P}}_{{\mathbb {T}},{\mathcal {L}}}^d \rightarrow {\mathbb {R}}$ is a *valuation*, that is,
  $$\begin{aligned} c_i^b(P \cup Q) + c_i^b(P \cap Q) = c_i^b(P) + c_i^b(Q) , \end{aligned}$$
  for all $P,Q \in {\mathcal {P}}_{{\mathbb {T}},{\mathcal {L}}}^d$ such that $P \cup Q, P \cap Q \in {\mathcal {P}}_{{\mathbb {T}},{\mathcal {L}}}^d$.

#### Proof

(i): We use the relationship between the Ehrhart polynomials of a tropical lattice polytope and its $\exp _b$-image in Remark 3.7. More precisely, we have

$$\begin{aligned} {\mathfrak {L}}_{\lambda \odot P}^b(k)&= \#\left( (k \odot \lambda \odot P) \cap \Gamma _b^d\right) = \#\left( b^k \cdot \exp _b(\lambda \odot P) \cap {\mathbb {Z}}^d\right) \\&= \#\left( b^k \cdot b^\lambda \cdot \exp _b(P) \cap {\mathbb {Z}}^d\right) = {\mathfrak {L}}_{P}^b(k+\lambda ). \end{aligned}$$

Since ${\mathfrak {L}}_{\lambda \odot P}^b(k) = \sum _{i=0}^d c_i^b(\lambda \odot P) (b^k)^i$ and ${\mathfrak {L}}_{P}^b(k+\lambda ) = \sum _{i=0}^d c_i^b(P)(b^{k+\lambda })^i$, the claimed identity follows by comparing coefficients.

(ii): Clearly, the counting function $P \mapsto \#\,\left( (k \odot P) \cap \Gamma _b^d\right)$ is a valuation, for every fixed $k \in {\mathbb {Z}}_{\ge 0}$. Therefore,

$$\begin{aligned} {\mathfrak {L}}_{P \cup Q}^b(k) + {\mathfrak {L}}_{P \cap Q}^b(k) = {\mathfrak {L}}_{P}^b(k) + {\mathfrak {L}}_{Q}^b(k) , \end{aligned}$$

and since every involved summand is a polynomial in $b^k$ of degree *d*, the claim follows by comparing coefficients. $\square$

#### Example 3.17

**Fig. 6:**
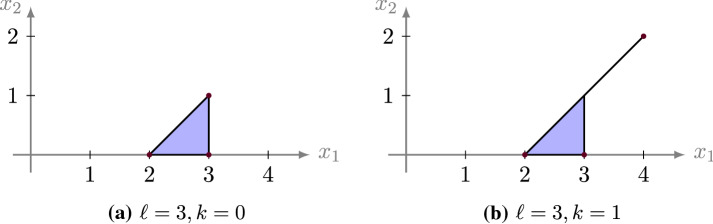
The tropical lattice polygons in Example 3.17

For $\ell \in {\mathbb {Z}}_{>0}$ and $k \in {\mathbb {Z}}_{\ge 0}$, let $$M = \left( \begin{array}{ccc} \ell -1 &{} \ell &{} k+\ell \\ 0 &{} 0 &{} k+1 \end{array} \right) $$ and consider the tropical lattice polygon $P = {{\,\mathrm{tconv}\,}}(M)$ (see Fig. 6). We aim to compute its first tropical Ehrhart coefficient $c_1^b(P)$.

Note, that *P* decomposes into the alcoved triangle $T=\Delta ((\ell -1,0)^\intercal )$ and the segment $S=[(\ell ,1)^\intercal ,(k+\ell ,k+1)^\intercal ]$, which itself is decomposed into the alcoved segments $S_j = (\ell +j-1,j)^\intercal + [{\mathbf {0}},{\mathbf {1}}]$, for $1 \le j \le k$. Hence, by the valuation property in Proposition 3.16, and the fact that the occurring intersections are zero-dimensional, we get by Lemma 3.11 and Example 3.13

$$\begin{aligned} c_1^b(P)&= c_1^b(T) + c_1^b(S_1) + \cdots + c_1^b(S_k)\\&= \frac{1}{2}(b-1)(b^{\ell -1}+2) + (b-1)(b+\cdots +b^k). \end{aligned}$$

## Tropical volume from tropical lattice points

### A novel concept of tropical volume

Motivated by the Ehrhart polynomials from the last section, we introduce a volume notion for tropical polytopes. After stating the definition, we explain its derivation through a two stage limit process: We use the discretization of volume by lattice points and let the fineness parameter of the tropical lattice go to infinity. To get started, recall Definition 2.1 of the *d*-trunk as the tropical volume concept relies only on the *d*-trunk of a tropical polytope.

#### Definition 4.1

(*Tropical barycentric volume*) Let $P \subseteq {\mathbb {T}}^d$ be a tropical polytope. The *tropical barycentric volume* of *P* is defined as

$$\begin{aligned} {{\,\mathrm{tbvol}\,}}(P) := \max _{x \in {{\,\mathrm{Tr}\,}}_d(P)} (x_1+\cdots +x_d). \end{aligned}$$

The tropical barycentric volume has a particularly nice form if the tropical polytope is pure. For this, we need the notion of the *tropical barycenter*, which is the componentwise maximal point of a tropical polytope. This point exists, and it is moreover unique due to the definition of tropical convex combinations. Proposition 2.3 implies the following.

#### Proposition 4.2

The tropical barycentric volume is the sum of the coordinates of the tropical barycenter of its *d*-trunk. In particular, the tropical barycentric volume of a pure tropical polytope is the sum of the coordinates of its tropical barycenter.

This observation also explains our choice to call ${{\,\mathrm{tbvol}\,}}(\cdot )$ the tropical *barycentric* volume.

#### Example 4.3

The tropical unit cube in ${\mathbb {T}}^d$ is given as the Cartesian product $[-\infty ,0]^d$. It can be written as the tropical convex hull of the points $(-\infty ,\ldots ,-\infty )^\intercal$, $(0,-\infty ,\ldots ,-\infty )^\intercal , \ldots , (-\infty ,\ldots ,-\infty ,0)^\intercal$.

Its tropical barycentric volume equals 0, the tropical multiplicative unit.

We now demonstrate how the tropical barycentric volume can be derived from a finer volume concept which relies on fixing the fineness parameter *b* of a tropical lattice. For the semiring $S_{(\max ,\cdot )}$, the Euclidean volume is well-behaved with respect to the arithmetic operations. As each tropical polytope $P \subseteq {\mathbb {T}}^d$ is the $\log _b$-image of a polytope over $S_{(\max ,\cdot )}$, this motivates the following.

#### Definition 4.4

(*Tropical* *b*-*volume*) The *tropical* *b*-*volume* ${{\,\mathrm{tbvol}\,}}^b(P)$ of *P* is the Euclidean volume ${{\,\mathrm{vol}\,}}(\exp _b(P))$.

This ties in with our deduction of tropical Ehrhart polynomials through the discretization of volume by lattice points. Using the polynomiality of the counting function $k \mapsto {\mathfrak {L}}_P^b(k)$ established in Sect. 3, we can easily build up an analogy to the classical setting: If $P \subseteq {\mathbb {R}}^d$ is a classical lattice polytope, that is, with respect to $(+,\cdot )$, and with Ehrhart polynomial $\#\,\left( k P \cap {\mathbb {Z}}^d \right) = \sum _{i=0}^d c_i(P) k^i$, then by properties of the Lebesgue-measure one obtains

$$\begin{aligned} {{\,\mathrm{vol}\,}}(P) = \lim _{k \rightarrow \infty } \frac{\#\,\left( k P \cap {\mathbb {Z}}^d \right) }{k^d} = c_d(P) . \end{aligned}$$

We have $k^d = \#\,\left( k \cdot [0,1)^d \cap {\mathbb {Z}}^d \right)$, that is, $k^d$ is the number of lattice points in the *k*th dilate of the standard fundamental cell of ${\mathbb {Z}}^d$. The tropicalization of this statement is given by

$$\begin{aligned} \#\,\left( \left( k \odot [-\infty ,0)^d\right) \cap \Gamma _b^d\right) = \#\,\left( [-\infty ,k)^d \cap \Gamma _b^d\right) = (b^k)^d . \end{aligned}$$

Thus writing ${\mathfrak {L}}_P^b(k) = \sum _{i=0}^d c_i^b(P) (b^k)^i$ for a *tropical* lattice polytope $P \subseteq {\mathbb {T}}^d$, we obtain

$$\begin{aligned} c_d^b(P) = \lim _{k \rightarrow \infty } \frac{\#\,\left( (k \odot P) \cap \Gamma _b^d\right) }{(b^k)^d} . \end{aligned}$$

In summary, we have proved the following statement.

#### Proposition 4.5

Let $P \subseteq {\mathbb {T}}^d$ be a tropical lattice polytope and let $b \in {\mathbb {N}}_{\ge 2}$. The *tropical* *b*-*volume* ${{\,\mathrm{tbvol}\,}}^b(P)$ of *P* is the leading coefficient $c_d^b(P)$ of its tropical Ehrhart polynomial ${\mathfrak {L}}_P^b(k)$.

#### Example 4.6

Consider the translated standard alcoved simplex $\Delta (a) \subseteq {\mathbb {T}}^d$, where $a \in {\mathbb {Z}}_{\ge 0}^d$. By Theorem 3.14, its tropical *b*-volume equals

$$\begin{aligned} {{\,\mathrm{tbvol}\,}}^b(\Delta (a)) = c_d^b(\Delta (a)) = (b-1)^d c_d(D_b^a \Delta ({\mathbf {0}})) = \tfrac{1}{d!} (b-1)^d b^{a_1+\cdots +a_d}. \end{aligned}$$

This example shows that the tropical *b*-volume of a tropical lattice polytope *P* equals the sum of ${{\,\mathrm{tbvol}\,}}^b(\Delta _\pi (a)) = \tfrac{1}{d!} (b-1)^d b^{a_1+\cdots +a_d}$, where $\Delta _\pi (a) \in {\mathcal {T}}_P$, for some $a \in {\mathbb {Z}}^d$ and some permutation $\pi \in S_d$. As a consequence ${{\,\mathrm{tbvol}\,}}^b(P)$, seen as a function of $b \in {\mathbb {N}}_{\ge 2}$, is a polynomial. Hence, applying the *logarithm-map*

$$\begin{aligned} {{\,\mathrm{Log}\,}}\,|f | := \lim _{b \rightarrow \infty } \log _b \,|f(b) | \end{aligned}$$

to ${{\,\mathrm{tbvol}\,}}^b(P)$, we arrive at a tropical volume concept for *P* which is independent of any additional parameter. The limit ${{\,\mathrm{Log}\,}}\,|f |$ does not exist for all functions $f:{\mathbb {N}}\rightarrow {\mathbb {R}}$; however, we only apply it to the rational functions $c_d^b(P)$ and $c_{d-1}^b(P)$ (cf. Lemma 5.6), which turn out to be polynomials.

#### Lemma 4.7

Let $P \subseteq {\mathbb {T}}^d$ be a tropical lattice polytope. Then

$$\begin{aligned} {{\,\mathrm{Log}\,}}\,|c_d^b(P) | = \max \{ a_1+\cdots +a_d + d : a \in {\mathbb {Z}}^d\text { such that } \Delta _\pi (a) \in {\mathcal {T}}_P \} . \end{aligned}$$

Note that the alcoved simplices $\Delta _\pi (a)$ appearing in the latter equation are full-dimensional.

Finally, we derive an expression for the limit of the leading coefficient ${{\,\mathrm{Log}\,}}\,|c_d^b(P) |$ of the tropical Ehrhart polynomial of *P* which is independent of the requirement of being a tropical *lattice* polytope.

#### Proposition 4.8

The limit ${{\,\mathrm{Log}\,}}\,|c_d^b(P) |$ for a tropical lattice polytope *P* equals ${{\,\mathrm{tbvol}\,}}(P)$.

#### Proof

Let $P \subseteq {\mathbb {T}}^d$ be a tropical lattice polytope. The *d*-trunk of *P* is the union of all full-dimensional alcoved simplices $\Delta _\pi (a) \in {\mathcal {T}}_P$. In the following, we use the compact and more convenient notation ${\mathbf {1}}^\intercal x = x_1+\cdots +x_d$. The maximal point with respect to the linear functional ${\mathbf {1}}$ of such a simplex $\Delta _\pi (a)$ is given by $a + {\mathbf {1}}$, so its coordinate sum equals $a_1+\cdots +a_d+d = {\mathbf {1}}^{\intercal }a + d$. The claim follows by observing that the maximal point of ${{\,\mathrm{Tr}\,}}_d(P)$ is the maximal point of a suitable simplex $\Delta _\pi (a)$. $\square$

This last observation completes the two stage limit process that led us to define the tropical barycentric volume as in Definition 4.1.

With the developed notation, we can state the tropical version of the classical *Pick’s Theorem*. It relates the volume and the number of lattice points in a lattice polygon $Q \subseteq {\mathbb {R}}^2$ by (cf. [6, Ch. 2.6])

4 $$\begin{aligned} \#\,\left( Q \cap {\mathbb {Z}}^2\right) = {{\,\mathrm{vol}\,}}(Q) + \tfrac{1}{2}\#\,\left( \partial Q \cap {\mathbb {Z}}^2\right) + 1. \end{aligned}$$

The symbol $\partial Q$ denotes the boundary of the polygon *Q*. Similar to the tropical barycentric volume, the tropical analog of (4) is an asymptotic version of the classical one on *b*-lattices. It follows from (4) by applying the Logarithm map to Theorem 3.6 and using Proposition 4.8.

#### Proposition 4.9

If $P \subseteq {\mathbb {T}}^2$ is a tropical lattice polygon, then

$$\begin{aligned} {{\,\mathrm{Log}\,}}\,|\#\,\left( P \cap \Gamma ^2_b\right) | = {{\,\mathrm{tbvol}\,}}(P) \oplus {{\,\mathrm{Log}\,}}\,|c_1^b(P) | \oplus 0. \end{aligned}$$

For a more meaningful statement, we would need to have a geometric understanding of ${{\,\mathrm{Log}\,}}\,|c_1^b(P) |$; we refer to Sect. 5.4 for this matter.

### Properties of the tropical barycentric volume

We now collect basic properties of the tropical barycentric volume, exhibiting the close analogy to the Euclidean volume. To this end, we need to introduce some notation.

We write $r^{\odot k} := \underbrace{r \odot \cdots \odot r}_{k\text { times}}$ for tropical exponentiation. Furthermore, let ${\mathcal {P}}_{\mathbb {T}}^d$ be the family of tropical polytopes in ${\mathbb {T}}^d$. For $z \in {\mathbb {T}}^d$, we consider the diagonal matrix ${{\,\mathrm{diag}\,}}(z_1, \ldots , z_d) \in {\mathbb {T}}^{d \times d}$, and for an arbitrary permutation in the symmetric group $S_d$ on *d* elements, let $\Sigma$ be the corresponding tropical permutation matrix. The entries in these matrices that are not specified by $z \in {\mathbb {T}}^d$ or the corresponding permutation are $-\infty$, the tropical zero element. The matrices of the form ${{\,\mathrm{diag}\,}}(z_1,\ldots ,z_d) \odot \Sigma$ form the group $\Pi _d$ of *scaled permutation matrices*. For a matrix $A = (a_{ij}) \in {\mathbb {T}}^{d \times d}$ the *tropical determinant* is defined as ${{\,\mathrm{tdet}\,}}(A)=\bigoplus _{\pi \in S_d } \bigodot _{i=1}^d a_{i,\pi (i)}$. The subgroup ${\mathcal {R}}_d \subseteq \Pi _d$ consisting of the matrices with $\bigodot _{i=1}^d z_i = 0$, that is, those with tropical determinant equal to 0, is called the group of *tropical rotation matrices*.

#### Proposition 4.10

(i) *(Monotonicity)* For every $P,Q \in {\mathcal {P}}_{\mathbb {T}}^d$ with $P \subseteq Q$, we have
  $$\begin{aligned} {{\,\mathrm{tbvol}\,}}(P) \le {{\,\mathrm{tbvol}\,}}(Q). \end{aligned}$$
(ii) *(Valuation property)* ${{\,\mathrm{tbvol}\,}}: {\mathcal {P}}_{\mathbb {T}}^d \rightarrow {\mathbb {T}}$ is a *valuation* in the sense that
  $$\begin{aligned} {{\,\mathrm{tbvol}\,}}(P) \oplus {{\,\mathrm{tbvol}\,}}(Q) = {{\,\mathrm{tbvol}\,}}(P \cup Q) \oplus {{\,\mathrm{tbvol}\,}}(P \cap Q), \end{aligned}$$
  for every $P,Q \in {\mathcal {P}}_{\mathbb {T}}^d$ such that $P \cup Q, P \cap Q \in {\mathcal {P}}_{\mathbb {T}}^d$.
(iii) *(Rotation invariance)* For $M \in {\mathcal {R}}_d$ and $P \in {\mathcal {P}}_{\mathbb {T}}^d$, we have
  $$\begin{aligned} {{\,\mathrm{tbvol}\,}}(M \odot P) = {{\,\mathrm{tbvol}\,}}(P). \end{aligned}$$
(iv) *(Homogeneity)* For every $\lambda \in {\mathbb {T}}$ we have
  $$\begin{aligned} {{\,\mathrm{tbvol}\,}}(\lambda \odot P) = \lambda ^{\odot d} \odot {{\,\mathrm{tbvol}\,}}(P). \end{aligned}$$
(v) *(Non-singularity)* ${{\,\mathrm{tbvol}\,}}(P) = -\infty$ if and only if ${{\,\mathrm{Tr}\,}}_d(P) = \emptyset$.

We will prove a more general statement in Proposition 5.4.

#### Remark 4.11

Property (ii) in Proposition 4.10 actually holds in a stronger form. Indeed, ${{\,\mathrm{tbvol}\,}}: {\mathcal {P}}_{\mathbb {T}}^d \rightarrow {\mathbb {T}}$ is an *idempotent measure*, which means that $\max \left\{ {{\,\mathrm{tbvol}\,}}(P),{{\,\mathrm{tbvol}\,}}(Q)\right\} = {{\,\mathrm{tbvol}\,}}(P \cup Q)$. For a thorough investigation of idempotent measures, we refer the reader to Akian [1].

Further, a short calculation analogous to the proof of Proposition 5.4 shows that (iii) and (iv) can be unified as ${{\,\mathrm{tbvol}\,}}(M \odot P) = {{\,\mathrm{tdet}\,}}(M) \odot {{\,\mathrm{tbvol}\,}}(P)$, for every $M \in \Pi _d$.

The Euclidean volume ${{\,\mathrm{vol}\,}}(\cdot )$ is multiplicative with respect to taking Cartesian products, that is, for any ordinary polytopes $P \subseteq {\mathbb {R}}^d$ and $Q \subseteq {\mathbb {R}}^e$ we have ${{\,\mathrm{vol}\,}}(P \times Q) = {{\,\mathrm{vol}\,}}(P) \cdot {{\,\mathrm{vol}\,}}(Q)$. Again, the tropical barycentric volume ${{\,\mathrm{tbvol}\,}}(\cdot )$ exhibits an analogous behavior.

#### Proposition 4.12

Let $P \in {\mathcal {P}}_{\mathbb {T}}^d$ and $Q \in {\mathcal {P}}_{\mathbb {T}}^e$. Then, $P \times Q \in {\mathcal {P}}_{\mathbb {T}}^{d+e}$ and

$$\begin{aligned} {{\,\mathrm{tbvol}\,}}(P \times Q) = {{\,\mathrm{tbvol}\,}}(P) \odot {{\,\mathrm{tbvol}\,}}(Q). \end{aligned}$$

#### Proof

The fact that $P \times Q$ is a tropical polytope when *P* and *Q* are, was proven in [17, Thm. 2]. The claimed identity is based on the observation that taking the trunk commutes with taking Cartesian products, more precisely

5 $$\begin{aligned} {{\,\mathrm{Tr}\,}}_{d+e} (P \times Q) = {{\,\mathrm{Tr}\,}}_d (P) \times {{\,\mathrm{Tr}\,}}_e (Q). \end{aligned}$$

Indeed, for any face $F \in {\mathcal {F}}_{P \times Q}$ that is contained in the $(d+e)$-trunk, there is a face $G \in {\mathcal {F}}_{P \times Q}$ with $F \subseteq G$ and $\dim (G) = d+e$. Since every face of a product of polytopal complexes is a product of faces of the factors, we find $G_P \in {\mathcal {F}}_P$ and $G_Q \in {\mathcal {F}}_Q$ such that $G = G_P \times G_Q$, and since $\dim (G_P) + \dim (G_Q) = d+e$, we have $\dim (G_P)=d$ and $\dim (G_Q)=e$. Therefore, writing $F = F_P \times F_Q$ for some $F_P \in {\mathcal {F}}_P$ and $F_Q \in {\mathcal {F}}_Q$, we obtain $F_P \subseteq G_P$ and $F_Q \subseteq G_Q$ and thus $F \in {{\,\mathrm{Tr}\,}}_d(P) \times {{\,\mathrm{Tr}\,}}_d(Q)$. As all these arguments can be reversed, the relation (5) follows.

With this information, we now have

$$\begin{aligned} {{\,\mathrm{tbvol}\,}}(P \times Q)&= \max _{x \in {{\,\mathrm{Tr}\,}}_{d+e}(P \times Q)} {\mathbf {1}}^\intercal x = \max _{(y,z) \in {{\,\mathrm{Tr}\,}}_d(P) \times {{\,\mathrm{Tr}\,}}_e(Q)} {\mathbf {1}}^\intercal (y,z)\\&=\max _{y \in {{\,\mathrm{Tr}\,}}_d(P)} {\mathbf {1}}^\intercal y + \max _{z \in {{\,\mathrm{Tr}\,}}_e(Q)} {\mathbf {1}}^\intercal z = {{\,\mathrm{tbvol}\,}}(P) \odot {{\,\mathrm{tbvol}\,}}(Q). \end{aligned}$$

$$\square $$

#### Example 4.13

A *tropical prism* is the Cartesian product of a tropical polytope *P* and a 1-dimensional tropical polytope *L* in ${\mathbb {T}}$. As each 1-dimensional tropical polytope is pure, its tropical barycentric volume is just the coordinate of its maximal point. Writing $L = [p,q]$, we get ${{\,\mathrm{tbvol}\,}}(P \times L) = {{\,\mathrm{tbvol}\,}}(P) + q$.

### Tropical volume revisited

We compare our volume notion with the two volume concepts introduced by Depersin et al. in [15].

#### Second highest determinant

Recall that the tropical determinant of a matrix $A = (a_{ij}) \in {\mathbb {T}}^{d \times d}$ is defined as ${{\,\mathrm{tdet}\,}}(A)=\bigoplus _{\pi \in S_d } \bigodot _{i=1}^d a_{i,\pi (i)}$. Given a permutation $\sigma \in S_d$, we further write ${{\,\mathrm{tdet}\,}}^\sigma (A) = \bigoplus _{\pi \in S_d \setminus \{\sigma \}} \bigodot _{i=1}^d a_{i,\pi (i)}$. The tropical volume concept introduced in [15] can then be defined by

6 $$\begin{aligned} {{\,\mathrm{tvol}\,}}(A) = |{{\,\mathrm{tdet}\,}}(A) - {{\,\mathrm{tdet}\,}}^\sigma (A) | , \end{aligned}$$

where $\sigma \in S_d$ is a permutation at which ${{\,\mathrm{tdet}\,}}(A)$ is attained. Observe that this is a volume notion for matrices. For the sake of distinction, we call ${{\,\mathrm{tvol}\,}}(A)$ the *tropical determinantal volume* of *A*. This notion is motivated from an ‘energy gap’ in statistical physics used in [31]. As described in [15], the tropical determinantal volume is non-singular in the sense that ${{\,\mathrm{tvol}\,}}(A) = 0$ if and only if $P={{\,\mathrm{tconv}\,}}(A)$ is contained in a tropical hyperplane, and thus, if and only if ${{\,\mathrm{tbvol}\,}}(P) = -\infty$.

A property that distinguishes ${{\,\mathrm{tvol}\,}}(\cdot )$ from ${{\,\mathrm{tbvol}\,}}(\cdot )$ is that the former is translation invariant in the classical sense, that is, if we write $v+A$ for the matrix that arises from *A* after adding the vector $v \in {\mathbb {R}}^d$ to each column of *A*, then ${{\,\mathrm{tvol}\,}}(v+A) = {{\,\mathrm{tvol}\,}}(A)$. Hence, the homogeneity of ${{\,\mathrm{tbvol}\,}}(\cdot )$ described in Proposition 4.10 (iv) shows that the two volume concepts are incomparable.

Another difference with ${{\,\mathrm{tbvol}\,}}(\cdot )$ is that the tropical determinantal volume is only defined for a quadratic matrix. We thus discuss potential extensions of ${{\,\mathrm{tvol}\,}}(\cdot )$ to rectangular matrices. The metric quantities in Definitions 4.1 and 4.14 below are extended from a local measure to a global measure by taking a maximum, over points or submatrices. Applying this idea to ${{\,\mathrm{tvol}\,}}(\cdot )$ suggests to extend it to rectangular matrices $A \in {\mathbb {T}}^{d \times m}$ with $d \le m$ by setting

7 $$\begin{aligned} {{\,\mathrm{tvol}\,}}_{\text {max-sub}}(A) = \max _{J \in \left( {\begin{array}{c}[m]\\ d\end{array}}\right) } {{\,\mathrm{tvol}\,}}(A_J) , \end{aligned}$$

where $A_J$ is the submatrix of *A* with columns indexed by the elements in *J*. This definition keeps the desirable property that the tropical determinantal volume is zero if and only if the tropical convex hull is lower-dimensional.

In the study of tropical principal component analysis, the notion ${{\,\mathrm{tvol}\,}}(\cdot )$ is also discussed in [37, § 3.1]. The authors prove that the following notion also extends the tropical determinantal volume to rectangular matrices, but in terms of a sum of tropical distances:

8 $$\begin{aligned} {{\,\mathrm{tvol}\,}}_{\text {best-fit}}(A) = \min _{z \in {\mathbb {T}}^d} \sum _{j \in [m]} \,\hbox {d}_{\mathrm{Tr}} ({\mathcal {H}}(z),A_{\{j\}}) . \end{aligned}$$

Here,

$$\begin{aligned} {\mathcal {H}}(z) = \left\{ x \in {\mathbb {T}}^d : \exists \,i \ne j\text { such that } x_i + z_i = x_j + z_j \ge x_{\ell } + z_{\ell }, \forall \ell \in [d] \right\} \end{aligned}$$

is the tropical hyperplane defined by *z*, and

$$\begin{aligned} \hbox {d}_{\mathrm{Tr}}(v,w) = \max \left\{ |v_i - w_i - v_j + w_j| :1 \le i,j \le d\right\} \end{aligned}$$

is the generalized Hilbert projective metric (cf. [13]).

#### Tropical dequantized volume

The next concept introduced in [15] is the maximal tropical minor among the vertices. It arose from the dequantization of the Euclidean volume of polytopes over Puiseux series associated with a tropical polytope.

##### Definition 4.14

([15, Thm. 13]) For a matrix $M \in {\mathbb {T}}^{d \times m}$, the *tropical (upper) dequantized volume* of *M* is defined by

9 $$\begin{aligned} {{\,\mathrm{qtvol}\,}}^+(M) = \max _{J \in \left( {\begin{array}{c}[m]\\ d\end{array}}\right) } {{\,\mathrm{tdet}\,}}(M_J) . \end{aligned}$$

The idea behind this formula is that the volume is essentially dominated by the maximal determinant of a simplex contained in a polytope.

It turns out that the tropical dequantized volume is an upper bound on the tropical barycentric volume. This inequality is a special case of Theorem 5.12 that we prove later.

##### Theorem 4.15

Let $M \in {\mathbb {T}}{\mathbb {N}}^{d \times m}$ and let $P = {{\,\mathrm{tconv}\,}}(M)$ be the corresponding tropical lattice polytope. Then,

$$\begin{aligned} {{\,\mathrm{tbvol}\,}}(P) \le {{\,\mathrm{qtvol}\,}}^+(M). \end{aligned}$$

**Fig. 7:**
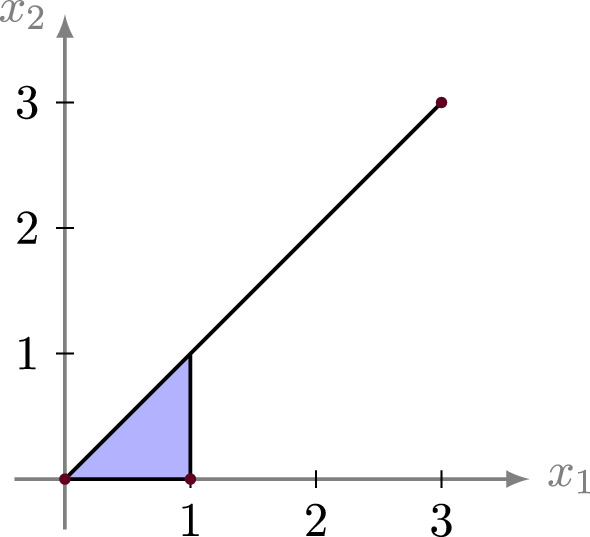
A tropical lattice polygon *P* with ${{\,\mathrm{tbvol}\,}}(P) = 2$ and ${{\,\mathrm{qtvol}\,}}^+(P) = 4$

There are examples for which this inequality is arbitrarily far from equality. For instance, for $\ell \in {\mathbb {Z}}_{>0}$, consider the matrix $$M = \left( \begin{array}{ccc} 0 &{} 1 &{} \ell \\ 0 &{} 0 &{} \ell \end{array} \right) $$ and the corresponding tropical polytope $P = {{\,\mathrm{tconv}\,}}(M)$. We find that ${{\,\mathrm{tbvol}\,}}(P) = 2$, whereas ${{\,\mathrm{qtvol}\,}}^+(M) = \ell +1$. The case $\ell =3$ is depicted in Fig. 7.

A characterization of the equality case follows right from Definition 4.1.

##### Proposition 4.16

Let $M \in {\mathbb {T}}{\mathbb {N}}^{d \times m}$ and let $P = {{\,\mathrm{tconv}\,}}(M)$. Then

$$\begin{aligned} {{\,\mathrm{tbvol}\,}}(P) = {{\,\mathrm{qtvol}\,}}^+(M) \end{aligned}$$

if and only if the tropical barycenter of *P* is contained in ${{\,\mathrm{Tr}\,}}_d(P)$.

##### Corollary 4.17

For pure tropical lattices polytopes $P = {{\,\mathrm{tconv}\,}}(M)$, the quantities ${{\,\mathrm{qtvol}\,}}^+(M)$ and ${{\,\mathrm{tbvol}\,}}(P)$ agree.

Although the previous discussion shows that the tropical volume concepts ${{\,\mathrm{tbvol}\,}}(\cdot )$ and ${{\,\mathrm{qtvol}\,}}^+(\cdot )$ are closely related, they are inherently different. For example, the multiplicativity of ${{\,\mathrm{tbvol}\,}}(\cdot )$ proved in Proposition 4.12 is not shared by ${{\,\mathrm{qtvol}\,}}^+(\cdot )$ in general: For $$M=\left( \begin{array}{ccc}0 &{} 1 &{} \ell \\ 0 &{} 0 &{} \ell \end{array}\right) $$ and $N=(0 \ 1)$, we have ${{\,\mathrm{qtvol}\,}}^+(M) = \ell + 1$ and ${{\,\mathrm{qtvol}\,}}^+(N) = 1$, while ${{\,\mathrm{qtvol}\,}}^+(M \times N) = 2 \ell + 1$. Here, $$M \times N := \left( \begin{array}{cccccc}0 &{} 1 &{} \ell &{} 0 &{} 1 &{} \ell \\ 0 &{} 0 &{} \ell &{} 0 &{} 0 &{} \ell \\ 0 &{} 0 &{} 0 &{} 1 &{} 1 &{} 1\end{array}\right) $$ represents the defining matrix of the product of the tropical polytopes ${{\,\mathrm{tconv}\,}}(M) \subseteq {\mathbb {T}}^2$ and ${{\,\mathrm{tconv}\,}}(N) \subseteq {\mathbb {T}}$.

## Metric estimates for tropical polytopes

In this section, we investigate generalizations of ${{\,\mathrm{tbvol}\,}}(\cdot )$ and ${{\,\mathrm{qtvol}\,}}^+(\cdot )$ to lower-dimensional quantities. Our definition of the tropical barycentric *i*-volumes below is mainly motivated by Theorem 5.7 and the discussion in Sect. 5.4, which aim to explain the second highest tropical Ehrhart coefficient as a kind of discrete tropical surface area. Another motivation comes from the connection to tropical *i*-minors that naturally extends Theorem 4.15 and suggests an estimate on all tropical Ehrhart coefficients (Conjecture 5.14) that has no counterpart in classical Ehrhart theory. Finally, we propose an analogy of the tropical barycentric *i*-volumes to the intrinsic volumes in convex geometry.

### Lower-dimensional tropical volumes and their properties

We start out with our definition of lower-dimensional tropical volume measures and then derive some basic properties.

#### Definition 5.1

(*Tropical barycentric* *i*-*volumes*) Let $P \subseteq {\mathbb {T}}^d$ be a tropical polytope and let $i \in [d]$. We define the *tropical upper barycentric* *i*-*volume* and the *tropical lower barycentric* *i*-*volume* of *P* by

$$\begin{aligned} {{\,\mathrm{tbvol}\,}}_i^+(P)&:= \max _{x \in {{\,\mathrm{Tr}\,}}_i(P)} \,\max \left\{ v^\intercal x : v \in \{0,1\}^d, {\mathbf {1}}^\intercal v = i \right\} \quad \text {and}\\ {{\,\mathrm{tbvol}\,}}_i^-(P)&:= \max _{x \in {{\,\mathrm{Tr}\,}}_i(P)} \,\min \left\{ v^\intercal x : v \in \{0,1\}^d, {\mathbf {1}}^\intercal v = i \right\} , \end{aligned}$$

respectively.

#### Example 5.2

We consider again the tropical polytope *P* from Example 2.2. As *P* is 2-dimensional, we get

$$\begin{aligned} {{\,\mathrm{tbvol}\,}}_4^+(P) = {{\,\mathrm{tbvol}\,}}_4^-(P) = {{\,\mathrm{tbvol}\,}}_3^+(P) = {{\,\mathrm{tbvol}\,}}_3^-(P) = -\infty . \end{aligned}$$

Using the explicitly given pseudovertices, we obtain ${{\,\mathrm{tbvol}\,}}_2^+(P) = 18$ (attained at each point of the 2-trunk), ${{\,\mathrm{tbvol}\,}}_2^-(P) = 2$ (attained at *p* and *q*), ${{\,\mathrm{tbvol}\,}}_1^+(P) = 9$ (attained at each point of the 1-trunk), ${{\,\mathrm{tbvol}\,}}_1^-(P) = 9$ (attained at *r*).

When we write ${{\,\mathrm{tbvol}\,}}_i^\pm (\cdot )$, we refer to both the upper and the lower tropical barycentric *i*-volume simultaneously. Each tropical barycentric *i*-volume comes with its own natural properties analogous to those of ${{\,\mathrm{tbvol}\,}}(\cdot )$ stated in Proposition 4.10. For the rotation invariance, we need the following refined subsets of scaled permutation matrices (see Sect. 4.2):

$$\begin{aligned} {\mathcal {R}}_{d,i}^+ := \left\{ {{\,\mathrm{diag}\,}}(z_1,\ldots ,z_d) \odot \Sigma : \max \big \{ v^\intercal z : v \in \{0,1\}^d, {\mathbf {1}}^\intercal v = i \big \} = 0 \right\} \subseteq \Pi _d, \end{aligned}$$

and

$$\begin{aligned} {\mathcal {R}}_{d,i}^- := \left\{ {{\,\mathrm{diag}\,}}(z_1,\ldots ,z_d) \odot \Sigma : \min \big \{ v^\intercal z : v \in \{0,1\}^d, {\mathbf {1}}^\intercal v = i \big \} = 0 \right\} \subseteq \Pi _d. \end{aligned}$$

We retrieve ${\mathcal {R}}_d = {\mathcal {R}}_{d,d}^\pm$ as a special case. We are not aware of a classical analog of ${\mathcal {R}}_{d,i}^{\pm }$.

#### Example 5.3

These subsets do not necessarily form a group for $i < d$ as the product

$$\begin{aligned} \begin{pmatrix} -\infty &{}\quad -\infty &{}\quad 2 \\ -3 &{}\quad -\infty &{}\quad -\infty \\ -\infty &{}\quad -2 &{}\quad -\infty \end{pmatrix} \odot \begin{pmatrix} -1 &{}\quad -\infty &{} \quad -\infty \\ -\infty &{}\quad -2 &{}\quad -\infty \\ -\infty &{}\quad -\infty &{}\quad 1 \end{pmatrix} = \begin{pmatrix} -\infty &{}\quad -\infty &{}\quad 3 \\ -4 &{}\quad -\infty &{}\quad -\infty \\ -\infty &{}\quad -4 &{} \quad -\infty \end{pmatrix} \not \in {\mathcal {R}}_{3,2}^+ \end{aligned}$$

shows. A typical matrix in ${\mathcal {R}}_{3,1}^-$ is

$$\begin{aligned} \begin{pmatrix} 0 &{}\quad -\infty &{}\quad -\infty \\ -\infty &{}\quad -\infty &{}\quad 4 \\ -\infty &{}\quad 5 &{}\quad -\infty \end{pmatrix} . \end{aligned}$$

Since ${{\,\mathrm{tbvol}\,}}_d^\pm (P) = {{\,\mathrm{tbvol}\,}}(P)$, the proof of the following properties also proves Proposition 4.10.

#### Proposition 5.4

(i) *(Monotonicity)* For every $P, Q \in {\mathcal {P}}_{\mathbb {T}}^d$ with $P \subseteq Q$, we have
  $$\begin{aligned} {{\,\mathrm{tbvol}\,}}_i^\pm (P) \le {{\,\mathrm{tbvol}\,}}_i^\pm (Q). \end{aligned}$$
(ii) *(Idempotency)* For every $P,Q \in {\mathcal {P}}_{\mathbb {T}}^d$ such that $P \cup Q \in {\mathcal {P}}_{\mathbb {T}}^d$, we have
  $$\begin{aligned} {{\,\mathrm{tbvol}\,}}_i^\pm (P) \oplus {{\,\mathrm{tbvol}\,}}_i^\pm (Q) = {{\,\mathrm{tbvol}\,}}_i^\pm (P \cup Q). \end{aligned}$$
(iii) *(Rotation invariance)* For every $P \in {\mathcal {P}}_{\mathbb {T}}^d$ and every $M \in {\mathcal {R}}_{d,i}^\pm$, we have
  $$\begin{aligned} {{\,\mathrm{tbvol}\,}}_i^\pm (M \odot P) = {{\,\mathrm{tbvol}\,}}_i^\pm (P). \end{aligned}$$
(iv) *(Homogeneity)* For every $\lambda \in {\mathbb {T}}$ we have
  $$\begin{aligned} {{\,\mathrm{tbvol}\,}}_i^\pm (\lambda \odot P) = \lambda ^{\odot i} \odot {{\,\mathrm{tbvol}\,}}_i^\pm (P). \end{aligned}$$
(v) *(Non-singularity)* ${{\,\mathrm{tbvol}\,}}_i^\pm (P) = -\infty$ if and only if ${{\,\mathrm{Tr}\,}}_i(P) = \emptyset$.

#### Proof

(i): If $P \subseteq Q$, then ${{\,\mathrm{Tr}\,}}_i(P) \subseteq {{\,\mathrm{Tr}\,}}_i(Q)$. Thus ${{\,\mathrm{tbvol}\,}}_i^\pm (P) \le {{\,\mathrm{tbvol}\,}}_i^\pm (Q)$.

(ii): If $P \cup Q \in {\mathcal {P}}_{\mathbb {T}}^d$, then ${{\,\mathrm{Tr}\,}}_i(P \cup Q) = {{\,\mathrm{Tr}\,}}_i(P) \cup {{\,\mathrm{Tr}\,}}_i(Q)$ from which the claimed identity follows.

(iii): Let $M = {{\,\mathrm{diag}\,}}(z_1,\ldots ,z_d) \odot \Sigma \in {\mathcal {R}}_{d,i}^-$. By definition

$$\begin{aligned} {{\,\mathrm{tbvol}\,}}_i^-(M \odot P)&= \max _{x \in {{\,\mathrm{Tr}\,}}_i(\Sigma \odot P + z)} \,\min \left\{ v^\intercal x : v \in \{0,1\}^d, {\mathbf {1}}^\intercal v = i \right\} \\&= {{\,\mathrm{tbvol}\,}}_i^-(\Sigma \odot P) + \min \left\{ v^\intercal z : v \in \{0,1\}^d, {\mathbf {1}}^\intercal v = i \right\} \\&= {{\,\mathrm{tbvol}\,}}_i^-(P). \end{aligned}$$

The proof for ${{\,\mathrm{tbvol}\,}}_i^+$ and matrices $M \in {\mathcal {R}}_{d,i}^+$ is analogous.

(iv): By definition

$$\begin{aligned} {{\,\mathrm{tbvol}\,}}_i^-(\lambda \odot P)&= \max _{x \in {{\,\mathrm{Tr}\,}}_i(P + \lambda {\mathbf {1}})} \,\min \left\{ v^\intercal x : v \in \{0,1\}^d, {\mathbf {1}}^\intercal v = i \right\} \\&= {{\,\mathrm{tbvol}\,}}_i^-(P) + \lambda i. \end{aligned}$$

Again, the proof for ${{\,\mathrm{tbvol}\,}}_i^+$ is analogous.

(v): Immediate from the definition. $\square$

It is easy to check that, since ${{\,\mathrm{Tr}\,}}_1(P) = P$, we have

10 $$\begin{aligned} {{\,\mathrm{tbvol}\,}}_1^+(P) = \max _{1 \le j \le d} {{\,\mathrm{tbvol}\,}}(\pi _j(P)) , \end{aligned}$$

where $\pi _j:{\mathbb {R}}^d \rightarrow {\mathbb {R}}$ is the projection onto the *j*th coordinate. This raises the question whether the tropical upper barycentric *i*-volumes admit a tropical analog of the integral representation formula for the intrinsic volumes (or quermassintegrals) of an ordinary polytope (see [35] for definition and properties). Roughly speaking, these formulae show that the *i*th intrinsic volume is the average of the volumes of the *i*-dimensional projections of the given polytope (cf. [10, Thm. 19.3.2] for details). However, the tropical polytope discussed in Examples 2.2 and 5.2 shows that the straightforward generalization of (10) does not hold without further reasonable assumptions.

In this line of thought, we thus pose

#### Question 5.5

Let $P \subseteq {\mathbb {T}}^d$ be a tropical polytope. Is it true that if $i \in [d]$ is an index with ${{\,\mathrm{Tr}\,}}_i(P)=P$, that then

$$\begin{aligned} {{\,\mathrm{tbvol}\,}}_i^+(P) = \max _{J \in \left( {\begin{array}{c}[d]\\ i\end{array}}\right) } {{\,\mathrm{tbvol}\,}}(\pi _J(P)) , \end{aligned}$$

where $\pi _J:{\mathbb {R}}^d \rightarrow {\mathbb {R}}^{|J |}$ is the projection onto the coordinates indexed by *J* ?

An analogous result cannot hold for the tropical lower barycentric *i*-volumes. Even for $i=1$, the valid inequality

$$\begin{aligned} {{\,\mathrm{tbvol}\,}}_1^-(P) \le \min _{1 \le i \le d} {{\,\mathrm{tbvol}\,}}(\pi _i(P)) \end{aligned}$$

can be strict.

### Estimating the second highest tropical Ehrhart coefficient

In this part, we argue how the tropical barycentric $(d-1)$-volumes can be used to estimate the second highest tropical Ehrhart coefficient. To this end, let $Q \subseteq {\mathbb {R}}^d$ be an *m*-dimensional classical lattice polytope, with $m \le d$. The *relative volume* of *Q* is defined as

$$\begin{aligned} {{\,\mathrm{rvol}\,}}(Q) := \frac{{{\,\mathrm{vol}\,}}_m(Q)}{\det ({\mathbb {Z}}^d \cap {{\,\mathrm{aff}\,}}(Q))} = \lim _{t \rightarrow \infty } \frac{1}{t^m} \cdot \#\,\left( tQ \cap {\mathbb {Z}}^d \right) , \end{aligned}$$

where ${{\,\mathrm{vol}\,}}_m(Q)$ denotes the Lebesgue measure in the affine hull ${{\,\mathrm{aff}\,}}(Q)$ of *Q*. Of course, if $m=d$, then ${{\,\mathrm{rvol}\,}}(Q) = {{\,\mathrm{vol}\,}}(Q)$. Let us record a well-known result from Ehrhart theory (cf. [6, Sect. 5.4]): The highest nonvanishing coefficient of the Ehrhart polynomial $\#\,\left( kQ \cap {\mathbb {Z}}^d\right) = \sum _{i=0}^d c_i(Q) k^i$ of *Q* is $c_m(Q)$ and it equals the relative volume of *Q*, that is, $c_m(Q) = {{\,\mathrm{rvol}\,}}(Q)$.

The second highest tropical Ehrhart coefficient $c_{d-1}^b(P)$ of a tropical lattice polytope $P \subseteq {\mathbb {T}}^d$ admits a more convenient representation than the signed sum in Theorem 3.14. To this end, recall that a simplex in ${\mathcal {T}}_P$ is called *maximal* if it is not properly contained in another simplex of ${\mathcal {T}}_P$.

#### Lemma 5.6

Let $P \subseteq {\mathbb {T}}^d$ be a tropical lattice polytope. Then

$$\begin{aligned} c_{d-1}^b(P) = \sum _{\begin{array}{c} \Delta _\pi ^s(a) \in {\mathcal {T}}_P\\ \dim (\Delta _\pi ^s(a)) = d-1 \end{array}} \delta (\Delta _\pi ^s(a)) \cdot (b-1)^{d-1} {{\,\mathrm{rvol}\,}}(D_b^a\Delta ^s_\pi ({\mathbf {0}})), \end{aligned}$$

where $\delta (\Delta _\pi ^s(a)) = 1$, if $\Delta _\pi ^s(a)$ is maximal, $\delta (\Delta _\pi ^s(a)) = 0$, if $\Delta _\pi ^s(a) \subseteq \mathop {P}\limits ^{\circ }$, and $\delta (\Delta _\pi ^s(a)) = \frac{1}{2}$, otherwise.

#### Proof

Specializing Theorem 3.14 to $i=d-1$ and in view of the remarks above, we haveThe classical description of the second highest Ehrhart coefficient of a lattice polytope (cf. (16)) implies that for *d*-dimensional alcoved simplices $\Delta _\pi ^s(a) \in {\mathcal {T}}_P$, we have , where the sum runs over the facets *F* of . Each of these facets corresponds to a $(d-1)$-dimensional alcoved simplex in ${\mathcal {T}}_P$, and hence, it appears in the first part of the representation of $c_{d-1}^b(P)$.

More precisely, if the facet is contained in the interior $\mathop {P}\limits ^{\circ }$ of *P*, then it is a facet of exactly two *d*-dimensional alcoved simplices in ${\mathcal {T}}_P$, and so it doesn’t contribute at all to $c_{d-1}^b(P)$. If the facet *F* is not contained in the interior, then it is a facet of exactly one alcoved simplex and it contributes $\frac{1}{2} (b-1)^{d-1} {{\,\mathrm{rvol}\,}}(F)$ to $c_{d-1}^b(P)$. $\square$

Based on this representation, we can now prove that the tropical barycentric $(d-1)$-volumes bound the second highest tropical Ehrhart coefficient.

#### Theorem 5.7

If $P \subseteq {\mathbb {T}}^d$ is a tropical lattice polytope, then

$$\begin{aligned} {{\,\mathrm{tbvol}\,}}_{d-1}^-(P) \le {{\,\mathrm{Log}\,}}\,|c^b_{d-1}(P) | \le {{\,\mathrm{tbvol}\,}}_{d-1}^+(P). \end{aligned}$$

#### Proof

Our arguments are based on the representation of $c_{d-1}^b(P)$ given in Lemma 5.6. We start with the claimed lower bound. As a minimum of linear functions, the function

$$\begin{aligned} x \mapsto \min \left\{ v^\intercal x : v \in \{0,1\}^d, {\mathbf {1}}^\intercal v = d-1 \right\} \end{aligned}$$

attains its maximum over ${{\,\mathrm{Tr}\,}}_{d-1}(P)$ at a boundary point and thus on a $(d-1)$-dimensional alcoved simplex $\Delta _\pi ^s(a) \in {\mathcal {T}}_P$ that has a nonzero contribution to $c_{d-1}^b(P)$. Since the boundary of the $(d-1)$-trunk of *P* is triangulated by the closures of those $\Delta _\pi ^s(a)$, it suffices to show that for these simplices

11 $$\begin{aligned} {{\,\mathrm{Log}\,}}\,|{{\,\mathrm{rvol}\,}}(D_b^a\Delta ^s_\pi ({\mathbf {0}})) | + d-1&\ge \max _{x \in \Delta _\pi ^s(a)} \min \Big \{ v^\intercal x : v \in \{0,1\}^d, {\mathbf {1}}^\intercal v = d-1 \Big \}. \end{aligned}$$

First of all, by symmetry we only need to consider $\pi =id$. In order to compute the relative volume of $D_b^a\Delta ^s({\mathbf {0}})$, we note that there are indices $0 \le j_0< j_1< \cdots < j_{d-1} \le d$ such that the closure of $\Delta ^s({\mathbf {0}})$ is given by

$$\begin{aligned} \overline{\Delta ^s({\mathbf {0}})} = {{\,\mathrm{conv}\,}}\left\{ e_{[j_0]},e_{[j_1]},\ldots ,e_{[j_{d-1}]}\right\} , \end{aligned}$$

where $e_{[j]} = e_1 + \cdots + e_j$ and $e_{[0]} = {\mathbf {0}}$. The linear subspace parallel to the affine span of $D_b^a \Delta ^s({\mathbf {0}})$ is thus given by

$$\begin{aligned} L_b^a(s)&= {{\,\mathrm{lin}\,}}\left\{ D_b^a(e_{[j_1]} - e_{[j_0]}),D_b^a(e_{[j_2]} - e_{[j_0]}),\ldots ,D_b^a(e_{[j_{d-1}]} - e_{[j_0]}) \right\} \\&= {{\,\mathrm{lin}\,}}\left\{ D_b^a(e_{[j_1]} - e_{[j_0]}),D_b^a(e_{[j_2]} - e_{[j_1]}),\ldots ,D_b^a(e_{[j_{d-1}]} - e_{[j_{d-2}]}) \right\} . \end{aligned}$$

The determinant of the $(d-1)$-dimensional sublattice ${\mathbb {Z}}^d \cap {{\,\mathrm{aff}\,}}(D_b^a\Delta ^s({\mathbf {0}}))$ can be estimated by

12 $$\begin{aligned} \det \left( {\mathbb {Z}}^d \cap {{\,\mathrm{aff}\,}}(D_b^a\Delta ^s({\mathbf {0}}))\right) = \det \left( {\mathbb {Z}}^d \cap L_b^a(s)\right) \le \det \left( V^\intercal V\right) ^{\frac{1}{2}}, \end{aligned}$$

where $V \in {\mathbb {Z}}^{d \times (d-1)}$ is any matrix whose columns $\{v_1,\ldots ,v_{d-1}\}$ are linearly independent vectors from ${\mathbb {Z}}^d \cap L_b^a(s)$. Note that

$$\begin{aligned} D_b^a(e_{[j_l]} - e_{[j_{l-1}]}) = b^{a_{j_{l-1}+1}} e_{j_{l-1}+1} + \cdots + b^{a_{j_l}} e_{j_l} =: \overline{v_l}, \end{aligned}$$

so that $v_l := b^{-\min \{a_{j_{l-1}+1},\ldots ,a_{j_l}\}} \cdot \overline{v_l} \in {\mathbb {Z}}^d \cap L_b^a(s)$, for every $l=1,\ldots ,d-1$. Here we used that *P* is a tropical lattice polytope, and thus $a \in {\mathbb {Z}}^d_{\ge 0}$.

Before applying (12) to estimate the determinant of said sublattice, we observe that $(d-1)!\cdot {{\,\mathrm{vol}\,}}_{d-1}(D_b^a\Delta ^s({\mathbf {0}}))$ equals the $(d-1)$-volume of the parallelepiped spanned by $D_b^a(e_{[j_1]} - e_{[j_0]}),D_b^a(e_{[j_2]} - e_{[j_0]}),\ldots ,D_b^a(e_{[j_{d-1}]} - e_{[j_0]})$. This in turn equals the $(d-1)$-volume of the parallelepiped $Q_{d-1}$ spanned by $\overline{v_1},\ldots ,\overline{v_{d-1}}$.

We have, ${\overline{v}}_l^\intercal {\overline{v}}_k = 0$, for $l \ne k$, and ${\overline{v}}_l^\intercal {\overline{v}}_l = \sum _{r=j_{l-1}+1}^{j_l} b^{2a_r}$. Hence, ${\overline{V}}^\intercal {\overline{V}}$ is a diagonal matrix and evaluating its determinant gives the following formula that we record for later use

13 $$\begin{aligned} {{\,\mathrm{vol}\,}}_{d-1}(D_b^a \Delta ^s({\mathbf {0}}))&= \frac{1}{(d-1)!} \prod _{t=0}^{d-2} \left( \sum _{\ell =j_t+1}^{j_{t+1}} b^{2 a_\ell } \right) ^{\frac{1}{2}}. \end{aligned}$$

Now, using (12) for the matrix $V=(v_1,\ldots ,v_{d-1})$, we get

$$\begin{aligned} \det \left( {\mathbb {Z}}^d \cap L_b^a(s)\right)&\le \prod _{l=1}^{d-1} b^{-\min \{a_{j_{l-1}+1},\ldots ,a_{j_l}\}} \det ({\overline{V}}^\intercal {\overline{V}})^{\frac{1}{2}}\\&= b^{-\sum _{l=1}^{d-1} \min \{a_{j_{l-1}+1},\ldots ,a_{j_l}\}} {{\,\mathrm{vol}\,}}_{d-1}(Q_{d-1}). \end{aligned}$$

Putting things together we arrive at the following lower bound on the relative volume of $D_b^a\Delta ^s({\mathbf {0}})$:

$$\begin{aligned} {{\,\mathrm{rvol}\,}}(D_b^a\Delta ^s({\mathbf {0}})) = \frac{{{\,\mathrm{vol}\,}}_{d-1}(D_b^a\Delta ^s({\mathbf {0}}))}{\det ({\mathbb {Z}}^d \cap {{\,\mathrm{aff}\,}}(D_b^a\Delta ^s({\mathbf {0}})))} \ge \tfrac{1}{(d-1)!} \cdot b^{\sum _{l=1}^{d-1} \min \{a_{j_{l-1}+1},\ldots ,a_{j_l}\}}. \end{aligned}$$

Now, the map ${{\,\mathrm{Log}\,}}\,|\cdot |$ is monotone in the sense that ${{\,\mathrm{Log}\,}}\,|f | \ge {{\,\mathrm{Log}\,}}\,|g |$ whenever $|f(b) | \ge |g(b) |$ for all $b \in {\mathbb {N}}$. Therefore,

$$\begin{aligned} {{\,\mathrm{Log}\,}}\,|{{\,\mathrm{rvol}\,}}(D_b^a\Delta ^s({\mathbf {0}})) |&\ge {{\,\mathrm{Log}\,}}\,|\tfrac{1}{(d-1)!} \cdot b^{\sum _{l=1}^{d-1} \min \{a_{j_{l-1}+1},\ldots ,a_{j_l}\}} |\\&= \sum _{l=1}^{d-1} \min \{a_{j_{l-1}+1},\ldots ,a_{j_l}\}, \end{aligned}$$

and so for (11) it suffices to show that

14 $$\begin{aligned}&\sum _{l=1}^{d-1} \min \{a_{j_{l-1}+1},\ldots ,a_{j_l}\} + d-1 \nonumber \\&\quad \ge \max _{x \in \Delta ^s(a)} \min \left\{ v^\intercal x : v \in \{0,1\}^d, {\mathbf {1}}^\intercal v = d-1 \right\} . \end{aligned}$$

The maximum on the right hand side is attained at a vertex of $\Delta ^s(a)$, that is, at a point of the form $a + e_{[j_l]}$, $l=1,\ldots ,d-1$. It thus evaluates to

$$\begin{aligned}&\max _{l=1,\ldots ,d-1} \,\min \left\{ v^\intercal (a+e_{[j_l]}) : v \in \{0,1\}^d, {\mathbf {1}}^\intercal v = d-1 \right\} \\&= \min \left\{ v^\intercal (a+e_{[j_{d-1}]}) : v \in \{0,1\}^d, {\mathbf {1}}^\intercal v = d-1 \right\} . \end{aligned}$$

Since $j_0< \cdots < j_{d-1}$, this implies (14) and thus the claimed lower bound on ${{\,\mathrm{Log}\,}}\,|c_{d-1}^b(P) |$.

We now prove the upper bound. First note that the determinant of a $(d-1)$-dimensional sublattice *L* of ${\mathbb {Z}}^d$ is at least 1. Indeed, there always exists a nonzero vector $u \in {\mathbb {Z}}^d$ such that $\det (L) = \Vert u\Vert \ge 1$ (cf. [34, Cor. 1.3.5]).

Now, let us consider an alcoved simplex $\Delta _\pi ^s(a) \in {\mathcal {T}}_P$ with $\dim (\Delta _\pi ^s(a))=d-1$. Again by symmetry, we can concentrate on $\pi =id$. As before, we find indices $0 \le j_0< j_1< \cdots < j_{d-1} \le d$ such that

$$\begin{aligned} \overline{\Delta ^s({\mathbf {0}})} = {{\,\mathrm{conv}\,}}\left\{ e_{[j_0]},e_{[j_1]},\ldots ,e_{[j_{d-1}]}\right\} . \end{aligned}$$

The identity (13) yields

$$\begin{aligned} {{\,\mathrm{rvol}\,}}(D_b^a\Delta ^s({\mathbf {0}}))&= \frac{{{\,\mathrm{vol}\,}}_{d-1}(D_b^a\Delta ^s({\mathbf {0}}))}{\det ({\mathbb {Z}}^d \cap {{\,\mathrm{aff}\,}}(D_b^a\Delta ^s({\mathbf {0}})))} \le {{\,\mathrm{vol}\,}}_{d-1}(D_b^a\Delta ^s({\mathbf {0}}))\\&= \frac{1}{(d-1)!} \prod _{t=0}^{d-2} \left( \sum _{\ell =j_t+1}^{j_{t+1}} b^{2 a_\ell } \right) ^{\frac{1}{2}}. \end{aligned}$$

Therefore,

$$\begin{aligned}&{{\,\mathrm{Log}\,}}\,|(b-1)^{d-1} {{\,\mathrm{rvol}\,}}(D_b^a\Delta _\pi ^s({\mathbf {0}})) | \le d-1 + \sum _{t=0}^{d-2} \max \{a_{j_t+1},\ldots ,a_{j_{t+1}}\}\\&\quad \le \max _{x \in \Delta _\pi ^s(a)} \max \left\{ v^\intercal x : v \in \{0,1\}^d, {\mathbf {1}}^\intercal v = d-1\right\} \le {{\,\mathrm{tbvol}\,}}_{d-1}^+(P). \end{aligned}$$

Since ${{\,\mathrm{Log}\,}}\,|f+g | \le \max \{{{\,\mathrm{Log}\,}}\,|f |,{{\,\mathrm{Log}\,}}\,|g |\}$, the formula in Lemma 5.6 gives us

$$\begin{aligned} {{\,\mathrm{Log}\,}}\,|c_{d-1}^b(P) |&\le \max _{\begin{array}{c} \Delta _\pi ^s(a) \in {\mathcal {T}}_P\\ \dim (\Delta _\pi ^s(a)) = d-1 \end{array}} {{\,\mathrm{Log}\,}}\,|\delta (\Delta _\pi ^s(a)) \cdot (b-1)^{d-1} {{\,\mathrm{rvol}\,}}(D_b^a\Delta ^s_\pi ({\mathbf {0}})) | \\&\le {{\,\mathrm{tbvol}\,}}_{d-1}^+(P), \end{aligned}$$

finishing the proof. $\square$

#### Remark 5.8

Both inequalities in Theorem 5.7 can be strict. Indeed, consider the matrix $$M = \left( \begin{array}{ccc} \ell -1 &{} \ell &{} k+\ell \\ 0 &{} 0 &{} k+1 \end{array} \right) $$. By the computations in Example 3.17, the tropical lattice polygon $P = {{\,\mathrm{tconv}\,}}(M)$ has parameters

$$\begin{aligned} \left( {{\,\mathrm{tbvol}\,}}_1^-(P),{{\,\mathrm{Log}\,}}\,|c_1^b(P) |,{{\,\mathrm{tbvol}\,}}_1^+(P)\right) = \Big (k+1,\max \{\ell ,k+1\},k+\ell \Big ) . \end{aligned}$$

### Tropical *i*-minors

In this part, we aim to extend Theorem 4.15 in order to give an upper estimate for the tropical lower barycentric *i*-volume in terms of tropical analogs of *i*-minors of the defining matrix *M* of *P*.

#### Definition 5.9

(*Maximal tropical* *i*-*minor*) Let $M \in {\mathbb {T}}^{d \times m}$ be a tropical matrix and let $i \in \{1,2,\ldots ,\min \{d,m\}\}$. We define the *maximal tropical* *i*-*minor* of *M* as

$$\begin{aligned} {{\,\mathrm{tm}\,}}_i(M) := \max _{I \in \left( {\begin{array}{c}[d]\\ i\end{array}}\right) , J \in \left( {\begin{array}{c}[m]\\ i\end{array}}\right) } {{\,\mathrm{tdet}\,}}(M_{I,J}) , \end{aligned}$$

where $M_{I,J}$ is the $i\times i$ submatrix of *M* whose rows are indexed by *I* and whose columns are indexed by *J*.

For $i = d$, we recover the tropical dequantized volume from Sect. 4.3.2. We need a generalization of [15, Prop. 15] to all maximal tropical *i*-minors. In order to state it, we record that in [15] a matrix $M \in {\mathbb {T}}^{d \times m}$ is called *tropically sign-generic* if for each $$J \in \left( {\begin{array}{c}[m]\\ d\end{array}}\right) $$ all permutations attaining ${{\,\mathrm{tdet}\,}}(M_J)$ have the same sign.

#### Lemma 5.10

Let $A \in {\mathbb {T}}^{d \times m}$ and $B \in {\mathbb {T}}^{d \times n}$ be such that ${{\,\mathrm{tconv}\,}}(A) \subseteq {{\,\mathrm{tconv}\,}}(B)$. If there are $$I \in \left( {\begin{array}{c}[d]\\ i\end{array}}\right) $$ and $$J \in \left( {\begin{array}{c}[m]\\ i\end{array}}\right) $$ such that $A_{I,J}$ is tropically sign-generic and ${{\,\mathrm{tm}\,}}_i(A) = {{\,\mathrm{tdet}\,}}(A_{I,J})$, then ${{\,\mathrm{tm}\,}}_i(A) \le {{\,\mathrm{tm}\,}}_i(B)$.

#### Proof

By projecting onto the coordinate subspace of ${\mathbb {T}}^d$ indexed by *I*, we see that ${{\,\mathrm{tconv}\,}}(A_{I,J}) \subseteq {{\,\mathrm{tconv}\,}}(B_{I,[n]})$. Applying [15, Prop. 15] to the two matrices $A_{I,J}$ and $B_{I,[n]}$, we get ${{\,\mathrm{tm}\,}}_i(A_{I,J}) \le {{\,\mathrm{tm}\,}}_i(B_{I,[n]})$. Using ${{\,\mathrm{tm}\,}}_i(A) = {{\,\mathrm{tdet}\,}}(A_{I,J})$, this yields ${{\,\mathrm{tm}\,}}_i(A) \le {{\,\mathrm{tm}\,}}_i(B)$. $\square$

#### Lemma 5.11

Let $\pi \in S_d$, let $0 \le j_0< j_1< \cdots < j_i \le d$ be indices, and let $S \in {\mathbb {T}}^{d \times (i+1)}$ be the matrix whose columns are $e^\pi _{[j_l]}:= e_{\pi (1)} + \cdots + e_{\pi (j_l)}$, for $l=0,1,\ldots ,i$. Then, there are $$I \in \left( {\begin{array}{c}[d]\\ i\end{array}}\right) $$ and $$J \in \left( {\begin{array}{c}[i+1]\\ i\end{array}}\right) $$ such that ${{\,\mathrm{tm}\,}}_i(S) = {{\,\mathrm{tdet}\,}}(S_{I,J})$ and $S_{I,J}$ is tropically sign-generic.

#### Proof

First of all, the statement and in particular ${{\,\mathrm{tm}\,}}_i(S)$ is invariant under permutations of the rows of *S*. Thus, we may assume that $\pi =id$. Second, ${{\,\mathrm{tm}\,}}_i(S) = i$ and it is attained by the $i \times i$-matrix arising from *S* after deleting the first column and keeping the rows corresponding to $j_1,\ldots ,j_i$. More precisely, ${{\,\mathrm{tm}\,}}_i(S) = {{\,\mathrm{tdet}\,}}(S_{I,J})$ for $I=\{j_1,\ldots ,j_i\}$ and $J=[i+1] \setminus \{1\}$. Furthermore, $S_{I,J}$ is an upper triangular matrix with 1’s on the diagonal. Thus, ${{\,\mathrm{tdet}\,}}(S_{I,J})$ is uniquely attained by the identity permutation and so $S_{I,J}$ is tropically non-singular, and in particular sign-generic. $\square$

In view of (9) and the identity ${{\,\mathrm{tbvol}\,}}(P) = {{\,\mathrm{Log}\,}}\,|c^b_d(P) |$, the following extends Theorem 4.15 to all tropical lower barycentric *i*-volumes.

#### Theorem 5.12

Let $M \in {\mathbb {T}}{\mathbb {N}}^{d \times m}$ and let $P = {{\,\mathrm{tconv}\,}}(M)$ be the corresponding tropical lattice polytope. Then, for every $i \in [d]$, we have

$$\begin{aligned} {{\,\mathrm{tbvol}\,}}_i^-(P) \le {{\,\mathrm{tm}\,}}_i(M). \end{aligned}$$

#### Proof

The *i*-trunk of *P* is the union of all $(\ge i)$-dimensional alcoved simplices occurring in the covector decomposition of *P*. If ${{\,\mathrm{Tr}\,}}_i(P) = \emptyset$, then ${{\,\mathrm{tbvol}\,}}_i^-(P) = -\infty$ and there is nothing to prove. We thus assume otherwise, and we let $\Delta _\pi ^s(a) \subseteq {{\,\mathrm{Tr}\,}}_i(P)$ be an alcoved simplex with $\dim (\Delta _\pi ^s(a)) \ge i$. Of course, it suffices to show that

15 $$\begin{aligned} \max _{x \in \Delta _\pi ^s(a)} \,\min \left\{ v^\intercal x : v \in \{0,1\}^d, {\mathbf {1}}^\intercal v = i \right\}&\le {{\,\mathrm{tm}\,}}_i(M). \end{aligned}$$

The maximum on the left-hand side is attained at a boundary point of $\Delta _\pi ^s(a)$, so that we can assume without loss of generality that $\dim (\Delta _\pi ^s(a)) = i$. There are indices $0 \le j_0< j_1< \cdots < j_i \le d$ such that

$$\begin{aligned} \Delta _\pi ^s(a) = a + {{\,\mathrm{conv}\,}}\left\{ e^\pi _{[j_0]},e^\pi _{[j_1]},\ldots ,e^\pi _{[j_i]}\right\} , \end{aligned}$$

where $e^\pi _{[l]} = e_{\pi (1)} + \cdots + e_{\pi (l)}$. Let $S \in {\mathbb {T}}^{d \times (i+1)}$ be the matrix whose columns correspond to the $i+1$ vertices of $\Delta _\pi ^s(a)$.

Combining Lemmas 5.10, 5.11, and ${{\,\mathrm{tconv}\,}}(S) = \Delta _\pi ^s(a) \subseteq P = {{\,\mathrm{tconv}\,}}(M)$, we see that ${{\,\mathrm{tm}\,}}_i(S) \le {{\,\mathrm{tm}\,}}_i(M)$. Thus, for (15) it suffices to show

$$\begin{aligned} \max _{x \in \Delta _\pi ^s(a)} \,\min \left\{ v^\intercal x : v \in \{0,1\}^d, {\mathbf {1}}^\intercal v = i \right\}&\le {{\,\mathrm{tm}\,}}_i(S). \end{aligned}$$

To this end, we first observe that by symmetry we may assume that $\pi =id$ and that $j_0=0$. Moreover, the maximum on the left hand side is attained at the point , since every $x \in \Delta ^s(a)$ is coordinate-wise dominated by  and because the function $x \mapsto v^\intercal x$ is non-decreasing with respect to this partial order.

Now, the *r*th coordinate of  is given by , if $r \le j_i$, and , if $r > j_i$. Therefore,  for every $1 \le l \le i$. For  defined by  if and only if $r \in \{j_1,\ldots ,j_i\}$, we thus obtain

For $i=1$ there is a more direct argument that gives a stronger result and allows to drop the integrality assumption:

#### Proposition 5.13

Let $M \in {\mathbb {T}}^{d \times m}$ and let $P = {{\,\mathrm{tconv}\,}}(M)$ be the corresponding tropical polytope. Then

$$\begin{aligned} {{\,\mathrm{tbvol}\,}}_1^-(P) \le {{\,\mathrm{tbvol}\,}}_1^+(P) = {{\,\mathrm{tm}\,}}_1(M), \end{aligned}$$

and equality holds if and only if ${{\,\mathrm{tm}\,}}_1(M)\cdot {\mathbf {1}}$ is the tropical barycenter of *P*.

#### Proof

First of all, ${{\,\mathrm{tm}\,}}_1(M) = \max _{1 \le i \le d, 1 \le j \le m} M_{i,j}$ is just the maximal entry of *M*. Moreover, for every $x \in P={{\,\mathrm{tconv}\,}}(M)$ there are coefficients $\gamma _1,\ldots ,\gamma _m \in {\mathbb {T}}$ with $\bigoplus _{j=1}^m \gamma _j = 0$ and $x = \bigoplus _{j=1}^m \gamma _j \odot M_{\cdot ,j}$. Since also $P={{\,\mathrm{Tr}\,}}_1(P)$, we have

$$\begin{aligned} {{\,\mathrm{tbvol}\,}}_1^-(P)&= \max _{x \in P} \min _{1 \le i \le d} x_i\\&= \max _{\gamma _1 \oplus \cdots \oplus \gamma _m = 0} \min _{1 \le i \le d} \max \{\gamma _1 + M_{i,1},\ldots ,\gamma _m + M_{i,m}\}\\&\le \max _{\gamma _1 \oplus \cdots \oplus \gamma _m = 0} \max _{1 \le i \le d} \max \{\gamma _1 + M_{i,1},\ldots ,\gamma _m + M_{i,m}\} = {{\,\mathrm{tm}\,}}_1(M). \end{aligned}$$

Equality holds if and only if there exist coefficients $\gamma _1,\ldots ,\gamma _m \in {\mathbb {T}}$ with $\gamma _1 \oplus \cdots \oplus \gamma _m = 0$ such that

$$\begin{aligned} \min _{1 \le i \le d} \max \{\gamma _1 + M_{i,1},\ldots ,\gamma _m + M_{i,m}\} = \max _{1 \le i \le d, 1 \le j \le m} M_{i,j}. \end{aligned}$$

This happens if and only if each row $M_{i,\cdot }$ contains a maximal entry of *M*. The corresponding coefficients would just be $\gamma _1 = \ldots = \gamma _m = 0$. In other words, the tropical barycenter of *P* equals ${{\,\mathrm{tm}\,}}_1(M)\cdot {\mathbf {1}}$. $\square$

We conjecture that the maximal tropical *i*-minors also upper bound the corresponding tropical Ehrhart coefficients, and that the following analogous bound to Theorem 5.12 holds:

#### Conjecture 5.14

Let $M \in {\mathbb {T}}{\mathbb {N}}^{d \times m}$ and let $P = {{\,\mathrm{tconv}\,}}(M)$ be the corresponding tropical lattice polytope. Then, for $i \in [d]$, we have

$$\begin{aligned} {{\,\mathrm{Log}\,}}\,|c^b_i(P) | \le {{\,\mathrm{tm}\,}}_i(M). \end{aligned}$$

#### Example 5.15

For $\ell \ge 2$, consider the example $$M = \left( \begin{array}{ccc} 0 &{}\quad 0 &{}\quad \ell -1 \\ 0 &{}\quad 1 &{}\quad \ell -1 \end{array} \right) $$ again (see Fig. 7). Writing $P = {{\,\mathrm{tconv}\,}}(M)$, we have

$$\begin{aligned} {\mathfrak {L}}_P^b(k)&= \tfrac{1}{2}(b-1)^2 (b^k)^2 + \tfrac{1}{2} ( b^{\ell -1} + 2b - 3 ) (b^k) + 1. \end{aligned}$$

Thus, $${{\,\mathrm{Log}\,}}\,|c^b_2(P) | = 2 \le \ell = {{\,\mathrm{tm}\,}}_2(M)$$ and $${{\,\mathrm{Log}\,}}\,|c^b_1(P) | = \ell - 1 = {{\,\mathrm{tm}\,}}_1(M)$$.

### Tropical surface areas

We end this section with a few musings on reasonable surface area concepts for tropical polytopes that naturally evolve from our previous studies. For one, the tropical barycentric $(d-1)$-volumes may serve as surface areas. Let us thus define the *upper* and *lower tropical surface area* of a tropical polytope $P \subseteq {\mathbb {T}}^d$ as

$$\begin{aligned} {{\,\mathrm{tbsurf}\,}}^+(P) := {{\,\mathrm{tbvol}\,}}_{d-1}^+(P) \quad \text {and} \quad {{\,\mathrm{tbsurf}\,}}^-(P) := {{\,\mathrm{tbvol}\,}}_{d-1}^-(P), \end{aligned}$$

respectively.

On the other hand, the second highest Ehrhart coefficient of an ordinary lattice polytope $Q \subseteq {\mathbb {R}}^d$ is a kind of *discrete* surface area (cf. [6, Thm. 5.6]). More precisely, writing $\#\,\left( k Q \cap {\mathbb {Z}}^d\right) = \sum _{i=0}^d c_i(Q) k^i$, we have

16 $$\begin{aligned} c_{d-1}(Q) = \frac{1}{2} \sum _{F\text { a facet of }Q} \frac{{{\,\mathrm{vol}\,}}_{d-1}(F)}{\det ({\mathbb {Z}}^d \cap {{\,\mathrm{aff}\,}}(F))} = \frac{1}{2} \sum _{F\text { a facet of }Q} {{\,\mathrm{rvol}\,}}(F). \end{aligned}$$

In this spirit, we may call

$$\begin{aligned} {{\,\mathrm{Log}\,}}\,|c_{d-1}^b(P) | \end{aligned}$$

the *discrete tropical surface area* of a tropical lattice polytope $P \subseteq {\mathbb {T}}^d$. Also, the formula for $c_{d-1}^b(P)$ in Lemma 5.6 suggests this as a surface area concept.

Natural questions for future studies arise from these definitions. First of all, we may ask for an isoperimetric inequality for tropical polytopes. The precise question taking the homogeneity of the magnitudes into account is as follows:

#### Question 5.16

Are there constants $c_d^+,c_d^- \in {\mathbb {T}}$ only depending on the dimension *d*, such that

$$\begin{aligned} {{\,\mathrm{tbvol}\,}}(P)^{\odot (d-1)} \le c_d^\pm \odot {{\,\mathrm{tbsurf}\,}}^\pm (P)^{\odot d}, \end{aligned}$$

for every tropical polytope $P \subseteq {\mathbb {T}}^d$?

Depersin et al. [15] established an isodiametric inequality for tropical simplices with respect to the functional ${{\,\mathrm{tvol}\,}}(\cdot )$ discussed in Sect. 4.3.1 and obtained interesting families of tropical polytopes along the way. We thus ask

#### Question 5.17

Is there an interesting isodiametric inequality with respect to ${{\,\mathrm{tbvol}\,}}(\cdot )$?

Regarding discrete surface area measures, we remark that Bey et al. [8, Prop. 4.2] proved an isoperimetric type inequality for lattice polytopes $Q \subseteq {\mathbb {R}}^d$. It states that $$c_{d-1}(Q) \le \left( {\begin{array}{c}d+1\\ 2\end{array}}\right) {{\,\mathrm{vol}\,}}(Q)$$.

#### Question 5.18

Does there exist a discrete isoperimetric inequality relating ${{\,\mathrm{tbvol}\,}}(\cdot )$ and ${{\,\mathrm{Log}\,}}\,|c_{d-1}^b(P) |$?

## Computational aspects

A matrix $M \in {\mathbb {T}}^{r \times r}$ is called *non-singular* if the value of the tropical determinant is $\ne -\infty$ and attained exactly once or equivalently, the tropical determinantal volume ${{\,\mathrm{tvol}\,}}(M) \ne 0$ (see (6) in Sect. 4.3). The *tropical rank* ${{\,\mathrm{trk}\,}}(M)$ of a matrix $M \in {\mathbb {T}}^{d \times m}$ is the size of a largest non-singular square submatrix of *M*. This notion was introduced and studied by Develin et al. who prove in [16, Thm. 4.2] that the tropical rank equals the dimension of $P={{\,\mathrm{tconv}\,}}(M)$ seen as a polytopal complex.

Recall that by Theorem 3.14 there exists an *i*-dimensional element in ${\mathcal {F}}_P$, if the tropical Ehrhart coefficient $c_i^b(P)$ is nonvanishing. Together with Corollary 3.15 this readily implies

### Lemma 6.1

Let $M \in {\mathbb {T}}{\mathbb {N}}^{d \times m}$ and let $P = {{\,\mathrm{tconv}\,}}(M)$. Then

$$\begin{aligned} {{\,\mathrm{trk}\,}}(M) = \max \left\{ i : c_i^b(P) \ne 0 \right\} . \end{aligned}$$

Kim and Roush [30, Thm. 13] showed that deciding if ${{\,\mathrm{trk}\,}}(M) \ge k$ is NP-complete. Their proof shows that this is true even for 0/1-matrices and thus we conclude

### Theorem 6.2

Let $P \subseteq {\mathbb {T}}^d$ be a tropical lattice polytope. Deciding whether $\max \left\{ i : c_i^b(P) \ne 0 \right\} \ge k$ is in general NP-hard.

Deciding whether the tropical barycentric volume ${{\,\mathrm{tbvol}\,}}(P)={{\,\mathrm{Log}\,}}\,|c_d^b(P) |$ is nonvanishing is a supposedly easier problem. For example, if *P* is a pure tropical lattice polytope, then by Corollary 4.17, we have ${{\,\mathrm{tbvol}\,}}(P) = {{\,\mathrm{qtvol}\,}}^+(M)$. In this case, the latter quantity and thus ${{\,\mathrm{tbvol}\,}}(P)$ can be computed in time $O(m^3)$ as shown in [15]. On the other hand, this decision problem is equivalent to (a) checking non-singularity of the defining matrix *M*, (b) checking feasibility of a tropical linear program, and (c) deciding winning positions in mean-payoff games. All these decision problems lie in NP $\cap$ coNP (cf. [22, § 2.2]).

### Proposition 6.3

Computing the tropical barycentric volume ${{\,\mathrm{tbvol}\,}}(P)$ is at least as hard as checking feasibility of a tropical linear inequality system.

One way to compute the tropical barycentric volume in Definition 4.1 is via the explicit determination of the covector decomposition, see [26], involving a classical convex hull computation.

We propose another possibility which is closer to the computation of the tropical dequantized volume defined in Definition 4.14. For this, we start by considering a *tropical simplex*, namely the tropical convex hull of a $d \times (d+1)$ matrix $A \in {\mathbb {T}}^{d \times (d+1)}$. We let  arise from *A* by appending a zero-th row filled with tropical ones 0. With the Hungarian method, one can compute the permutation attaining the tropical determinant  in $O(d^3)$, see [11, § 1.6.4]. Using the dual variables and reordering the columns, we can assume that the tropical determinant  is attained at the identity permutation, that all entries on the diagonal are 0 and that all off-diagonal entries are non-positive. One can deduce from [11, Lem. 4.3.2] that the columns of the *Kleene star*  provide generators of the *d*-trunk of ${{\,\mathrm{tconv}\,}}(A)$, by appropriately scaling so that the zero-th row consists only of 0’s again. Computing the Kleene star takes again $O(d^3)$ time. In summary, we have

### Proposition 6.4

The *d*-trunk of a tropical simplex in ${\mathbb {T}}^d$ is a polytrope. Its tropical barycenter can be computed in time $O(d^3)$.

As a consequence, we get that the tropical barycentric volume of a tropical polytope can be computed in polynomial time $O(m^{d+1})$ if the dimension *d* is fixed, as we see in the next statement.

### Proposition 6.5

Let $M \in {\mathbb {T}}^{d \times m}$ and let $P = {{\,\mathrm{tconv}\,}}(M)$. The tropical barycentric volume ${{\,\mathrm{tbvol}\,}}(P)$ is the maximumwhere ${{\,\mathrm{bt}\,}}(S)$ denotes the tropical barycenter of the *d*-trunk of a tropical simplex $S \subseteq {\mathbb {T}}^d$. Moreover, ${{\,\mathrm{tbvol}\,}}(P)$ can be computed in time $$O(\left( {\begin{array}{c}m\\ d+1\end{array}}\right) \cdot d^3)$$.

### Proof

By the tropical Carathéodory theorem, the tropical convex hull of *M* is the union of the tropical simplices ${{\,\mathrm{tconv}\,}}(M_J)$, $$J \in \left( {\begin{array}{c}[m]\\ d+1\end{array}}\right) $$. We compute the tropical barycenter of each of these tropical simplices in $O(d^3)$ time by Proposition 6.4. $\square$

### Remark 6.6

One could consider the tropical barycentric volume ${{\,\mathrm{tbvol}\,}}(P)$ as a robust version of a transportation problem. The tropical dequantized volume is the generalization of a maximal matching problem, namely a transportation problem [15, Cor. 18]. The tropical barycentric volume is the solution of the transportation problem for its *d*-trunk, without the lower-dimensional parts. In this sense, it is more robust with respect to perturbations.

### Question 6.7

Let $P \subseteq {\mathbb {T}}^d$ be a tropical polytope.

(i) How fast can we compute ${{\,\mathrm{tbvol}\,}}(P)$?
(ii) What is the computational complexity of deciding ${{\,\mathrm{tbvol}\,}}(P) \ne -\infty$?

Note that computing the volume of an ordinary polytope is #P-hard ([18]).
